# Use of Machine Learning with Fused Spectral Data for Prediction of Product Sensory Characteristics: The Case of Grape to Wine

**DOI:** 10.3390/foods12040757

**Published:** 2023-02-09

**Authors:** Claire E. J. Armstrong, Jun Niimi, Paul K. Boss, Vinay Pagay, David W. Jeffery

**Affiliations:** 1 Australian Research Council Training Centre for Innovative Wine Production, The University of Adelaide, PMB 1, Glen Osmond, SA 5064, Australia; 2 School of Agriculture, Food and Wine, and Waite Research Institute, The University of Adelaide, PMB 1, Glen Osmond, SA 5064, Australia; 3 CSIRO Agriculture and Food, Locked Bag 2, Glen Osmond, SA 5064, Australia

**Keywords:** A-TEEM, CIELAB, chemometrics, regression, wine, extreme gradient boosting

## Abstract

Generations of sensors have been developed for predicting food sensory profiles to circumvent the use of a human sensory panel, but a technology that can rapidly predict a suite of sensory attributes from one spectral measurement remains unavailable. Using spectra from grape extracts, this novel study aimed to address this challenge by exploring the use of a machine learning algorithm, extreme gradient boosting (XGBoost), to predict twenty-two wine sensory attribute scores from five sensory stimuli: aroma, colour, taste, flavour, and mouthfeel. Two datasets were obtained from absorbance-transmission and fluorescence excitation-emission matrix (A-TEEM) spectroscopy with different fusion methods: variable-level data fusion of absorbance and fluorescence spectral fingerprints, and feature-level data fusion of A-TEEM and CIELAB datasets. The results for externally validated models showed slightly better performance using only A-TEEM data, predicting five out of twenty-two wine sensory attributes with R^2^ values above 0.7 and fifteen with R^2^ values above 0.5. Considering the complex biotransformation involved in processing grapes to wine, the ability to predict sensory properties based on underlying chemical composition in this way suggests that the approach could be more broadly applicable to the agri-food sector and other transformed foodstuffs to predict a product’s sensory characteristics from raw material spectral attributes.

## 1. Introduction

Consumer acceptability of food products largely depends on sensory properties associated with appearance, aroma, flavour, taste, and mouthfeel [1]. The most common method to evaluate a product’s sensory profile is to use a human sensory panel, which can be limiting due to participant variability and availability and the time required to collect and analyse the results, especially if panel training is required. Accordingly, effort has been dedicated to designing systems that can provide an objective evaluation of the sensory profile of food products without the need for human testers [2,3].

Choosing appropriate analytical techniques and methods for interrogating multivariable datasets is key to achieving the goal of predicting sensory properties from chemical composition. In recent times, progression in multivariate data analysis and the fusing of datasets originating from several instrumental sources have advanced the determination of food sensory properties based on chemical and physical data [4]. Bioelectronic sensors and their combinations provide an example of the kinds of systems that use data from multiple sources to predict food sensory properties. They have been designed to mimic the human response to food colour, aroma, and taste, but issues can arise with sensitivity, selectivity, interferences, and matrix effects when applied to real-world samples [3].

In contrast, food chemical data collected using a spectroscopic technique (often based on UV-Vis, fluorescence, or infrared) can be used to predict sensory properties [5,6,7,8], thus potentially offering fast, straightforward, selective, and sensitive solutions [4]. Fluorescence spectroscopy, which yields a molecular fingerprint of a sample, is particularly appealing because of its high sensitivity compared to other spectroscopic methods [9]. Using a spectrophotometer originally employed for water quality analysis, the absorbance-transmission and fluorescence excitation-emission matrix (A-TEEM) method [10] offers a powerful option, being used recently in conjunction with chemometrics and machine learning for wine authentication, modelling the sensory and chemical characteristics of wine, and chemotyping of cannabis [11,12,13,14,15,16]. A-TEEM spectral fingerprints are based on compounds containing a fluorophore or chromophore (i.e., UV and visible wavelengths), which in the case of foods and beverages includes aromatic amino acids, phenolic compounds, vitamins, and porphyrins (e.g., chlorophyll) [17,18]. With the additional benefit of UV and fluorescence capabilities, the approach could be considered an extension of an electronic eye, which usually involves only the visible light spectra and affords RGB or CIELAB colour values [2,19]. In terms of modelling approaches, feature or variable-level data fusion methods exist for combining datasets from multiple sources, which use either pre-processed features of models, such as principal components from principal component analysis (PCA), or variables that have been normalised prior to fusion. Research has shown significant improvements in model performance for classification and prediction problems using feature-level data fusion rather than variable-level data fusion or unfused spectra [2,20,21].

In combination with the apparent power of A-TEEM and chemometric modelling, grape and wine appeared to be an ideal model system for exploring complex chemical and sensory relationships between raw material and product. For quality wine production, grape berry tasting is the most common method used in the industry to examine the flavour and aroma potential of wine grapes prior to harvest. However, berry tasting has been shown to have varying results between vintages and grape varieties [22,23], and substantial transformations occur to convert grape juice into wine via a multitude of microbiological and chemical processes [24]. Adding further to the challenge, the human perception of sensory characteristics is partly due to perceptual interactions (including synergistic or antagonistic) between a broad range of volatile and nonvolatile compounds that can be present at vastly different concentrations [25]. Prediction of wine sensory scores from grape chemical data, a highly ambitious but significant goal, has been explored in a few previous studies using a range of chemical measurements or mid-infrared spectroscopy [26,27,28]. In those cases, sequential-orthogonalised partial least-squares (SO-PLS) regression, which can efficiently handle multi-dimensionality in data matrices, and PLS regression each resulted in low to moderate performance.

Seeking to improve on the previous research with an innovative approach, a gradient-boosted decision tree algorithm known as XGBoost [29] was deemed to be a suitable machine learning method that could be used with spectral data for the prediction of wine sensory properties. XGBoost is a non-linear algorithm and has previously been used to good effect for classification and regression modelling [11,12,13,14,15,16]. Thus, the aim of the present work was to conduct a proof-of-concept study involving the novel application of A-TEEM analysis coupled with XGBoost for the prediction of ratings (scores) for a diverse range of wine sensory attributes from five sensory stimuli–colour, aroma, flavour, taste, and mouthfeel–using Cabernet Sauvignon grape extract spectra collected from different regions over three vintages. Parallel factor analysis (PARAFAC) was applied to grape extract excitation-emission matrices (EEMs) to explore the underlying fluorophoric compound classes that characterised the different samples. Feature-level fusion of A-TEEM and CIELAB datasets, collected simultaneously with an Aqualog instrument, was used for the modelling of sensory data, with a comparison to models obtained from the A-TEEM dataset alone. Ultimately, this work intended to demonstrate the capabilities of a methodology that could be developed as a rapid approach for the prediction of sensory attribute ratings of various foods or beverages.

## 2. Materials and Methods

### 2.1. Grape and Wine Samples

Cabernet Sauvignon grape samples were collected from multiple vineyards within eight viticultural regions across South Australia (Figure S1 of the Supplementary Material) at the time of harvest in 2013, 2014, and 2015. Full details of the sampling regime can be found elsewhere [22]. Wines were produced during the respective vintage years from each parcel of fruit using the same vinification method each year, following a previously reported 50 kg small-lot winemaking procedure [22]. Briefly, grapes were destemmed, crushed, and mixed with 50 mg/L of SO_2_ prior to inoculation with *Saccharomyces cerevisiae* yeast EC1118 (300 mg/L) in a 60 L plastic pail. Alcoholic fermentations were inoculated on the second day with *Oenococcus oeni* (2 mg/L, Lalvin VP41, Lallemand S.A.S) to conduct malolactic fermentation. At the completion of alcoholic fermentation (≤2.0 g/L of sugar), wines were pressed off skins, and malolactic fermentation was completed (malic acid below 0.2 g/L) in 20 L stainless steel kegs. Free SO_2_ was adjusted to 40 mg/L, 4 g/L potassium bitartrate was added, and wines were cold settled at 0 °C for 21 days. Wines were racked off lees and filtered using a Colombo-Rover pump and Z6 cellulose pad filter. Bottling occurred under nitrogen gas into 375 mL screw cap bottles after adjusting free SO_2_ to 35–40 mg/L. Bottled wines were stored for three months at 15 °C prior to sensory analysis.

### 2.2. A-TEEM and CIELAB Data Collection

The number of grape homogenate samples per vintage was 25, except for 2013, which had 24 samples due to the exclusion of a vineyard in Barossa Valley because of an insufficient amount of grape homogenate remaining to complete the spectral analyses. This provided a total of 74 grape homogenates to be considered with the corresponding wine sensory data. Grape samples were frozen in liquid nitrogen, homogenised to a powder, and stored in a −80 °C freezer at the time of harvest in 2013, 2014, and 2015 [28], with the type of preparation being acceptable for analysing red grape compositional aspects [30,31]. Spectroscopic measurements were conducted in 2021 according to the following procedure. Samples were kept frozen using liquid nitrogen while 1 g of grape homogenate powder was weighed out into a 15 mL centrifuge tube. Three replicates of grape homogenate samples per vineyard were weighed, giving a total of 72 samples for 2013 and 75 each for 2014 and 2015. Grape homogenate extraction was achieved following the previously reported method [32]. Briefly, 10 mL of 50% aqueous ethanol solution (*v*/*v*) prepared from gradient grade ethanol for liquid chromatography (Sigma Aldrich, Castle Hill, NSW, Australia) was added to each 1 g aliquot, and tubes were shaken for 1 h at room temperature on an EOM5 Orbital Shaker (Ratek Instruments, Boronia, Victoria, Australia). The tubes were centrifuged for 10 min at 1200 g, and 100 µL of the supernatant from each was transferred to a 4 mL glass vial and diluted with 2900 µL of acidified 50% ethanol solution (adjusted to pH 2.0 with 1 M HCl solution prepared from 37% HCl, HPLC gradient grade, Sigma Aldrich, Castle Hill, NSW, Australia) to give a 30-fold dilution factor that obeys the linear Beer-Lambert concentration relationship [33]. The diluted solution was vortexed for 30 s, sonicated for 10 min, and transferred to a 10 mm × 10 mm quartz glass fluorescence cuvette (Hellma Group, Mülheim, Germany) containing a stir bar. A HORIBA Scientific Aqualog spectrophotometer (Aqualog-UV-800-C, Quark Photonics, Adelaide, SA, Australia) was used for absorbance and fluorescence spectral acquisition according to the previously reported method [11]. The CIE function of the Aqualog software (version 4.2, HORIBA Instruments Inc., Irvine, CA, USA) was used to calculate the CIELAB colour coordinates from absorbance values. The CIELAB colour parameters were C*ab (chroma, radial component of colour coordinate system), H*ab (hue angle, angular component of colour coordinate system), L* (lightness, from black to white), a* (green to red colour space), b* (blue to yellow colour space), with the addition of S* (saturation, equal to C* divided by L*) and Q* (defined as $((0.15\times L^{*}\times \log Y)+\left(0.6\times L^{*}\;\right))+40$, where Y is equal to the green tristimulus value). Spectroscopic measurements were carried out in duplicate.

### 2.3. Wine Sensory Profiling

Descriptive sensory analysis of wines was completed three months after bottling in 2013, 2014, and 2015 by previous authors using a 15-cm scale and trained participants [22,26]. Between 9–11 assessors participated in the evaluations throughout 2013–2015. Gender varied from one to four males and six to nine females in any given year, with ages ranging from 25 to 60 years. The assessors were not always the same across the three years, depending on their availability at the time of testing. However, all assessors had experience in descriptive sensory analysis and comprised a mixture of staff and postgraduate oenology students at the University of Adelaide, as well as ISO-screened panellists who had training in the discrimination and recognition of basic tastes and odours. Panellists gave informed consent before participating, and the study was approved by The University of Adelaide’s Human Research Ethics Committee (H-2014-057). The list of attributes used in the current study was compiled by matching up the consensus-based descriptors from the previous work [22,26] across different vintages, giving a sensory block with 22 attributes from five sensory stimuli: colour, aroma, flavour, taste, and mouthfeel.

### 2.4. PARAFAC Modelling

Grape homogenate EEMs were split into two separate datasets for PARAFAC Model 1 and PARAFAC Model 2. An excitation wavelength range of 250–500 nm and an emission wavelength range of 270–500 nm were used for Model 1, with excitation at 370–650 nm and emission at 500–750 nm used for Model 2. First-order (±16 nm) and second-order (±32 nm) Rayleigh filters were applied to the EEMs, and scatter was replaced with ‘missing values’. EEMs had non-negativity constraints applied to all three axes (intensity, and emission and excitation wavelengths). Spectra were normalised to an area of one for both PARAFAC models.

### 2.5. Data Pre-Processing and Fusion

To achieve combined A-TEEM data for each sample using the variable-level data fusion method, 3D EEMs were reshaped to 2D, and the absorbance and fluorescence data blocks were Pareto scaled and block scaled by the square root of the pooled variance before joining [34,35]. Prior to PCA for feature-level data fusion, data were split randomly into Train (378 samples) and Test (66 samples) datasets (85:15 split) with spectroscopy duplicates kept together. PCA was carried out on mean-centred A-TEEM data and standardised CIELAB data, and the principal components that explained ≥ 1.00% of the variance of each dataset were extracted and block scaled by the square root of the pooled variance before data fusion.

### 2.6. XGBoost Regression Modelling

Generalised least squares weighting (α = 0.2) or external parameter orthogonalisation (number of principal components = 3) for interfering signal (clutter) covariance removal was applied to the X-block (fused spectroscopic data), and Pareto scaling and mean-centring were applied to the Y-block (sensory data) prior to all XGBoost modelling. A grid-search of hyperparameters associated with the XGBoost algorithm [29] was set up (Table S1 of the Supplementary Material) using Train data only, and data compression of A-TEEM data was achieved using PLS regression. The number of PLS latent variables (LVs) used for data compression was limited to 2–6 for optimum modelling. Feature-level fused A-TEEM and CIELAB datasets were not compressed before modelling. Cross-validation (CV) on the Train dataset using the Venetian blinds method [36], with the number of data splits equal to ten and a blind thickness equal to one, was used to tune hyperparameters. Root mean square error (RMSE) of cross-validation (RMSECV) was used to select the optimal Train model, which was then applied after CV to the Test dataset.

### 2.7. Software

Data pre-processing, fusion (multiblock tool), PARAFAC, and XGBoost modelling were carried out using Solo software (version 9.0, Eigenvector Research, Inc., Manson, WA, USA). Data visualisation and PCA were achieved using R (R Foundation for Statistical Computing, Vienne, Austria) in RStudio version 2022.02.3 (RStudio Inc., Boston, MA, USA) with packages “ggplot2”, “sf”, “FactoMineR”, and “factoextra”.

## 3. Results and Discussion

### 3.1. PARAFAC Examination of EEM Data

**Figure 1:**
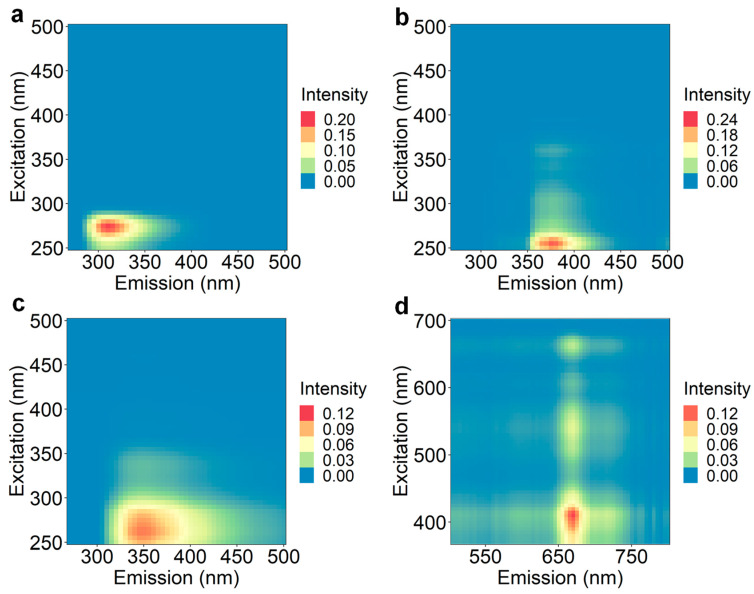
Grape extract excitation and emission maxima from PARAFAC showing (**a**) component 1, (**b**) component 2, and (**c**) component 3 for Model 1, and (**d**) component 1 for Model 2.

PARAFAC was applied to grape extract EEMs for exploratory analysis of the data to gain insight into the types of fluorophores that could explain the separation of different samples and relate to predictions of sensory attribute scores using the XGBoost algorithm. Two individual PARAFAC models were executed due to a low signal-to-noise ratio in the EEM region with excitation and emission greater than 500 nm. PARAFAC Model 1 included the EEM region with an excitation range of 250–500 nm and emission range of 270–500 nm (Figure 1a–c), and PARAFAC Model 2 included the excitation range of 370–700 nm and emission range of 500–800 nm (Figure 1d).

Three fluorophores were revealed by PARAFAC Model 1: component 1 had excitation/emission wavelength (λ_ex_/λ_em_) maxima at 275/310 nm (Figure 1a); component 2 had λ_ex_/λ_em_ maxima at 255/375 nm (Figure 1b); and component 3 had λ_ex_/λ_em_ maxima at 260/350 nm (Figure 1c). These PARAFAC components are similar to those previously reported for EEMs from Cabernet Sauvignon grape extracts [32]. On the basis of the λ_ex_/λ_em_, compound classes can be tentatively assigned to components 1 to 3 as monomeric catechins, caffeic/caftaric acid or procyanidin-related dimers, and phenolic acids (e.g., vanillic, gallic, and syringic acid), respectively. One component was identified within the EEM landscape for PARAFAC Model 2 with λ_ex_ maxima at 410 nm and λ_em_ maxima at 670 nm with a shoulder at 715 nm (Figure 1d); this was tentatively assigned to chlorophyll a or b. The corresponding core consistency values for PARAFAC Models 1 and 2 were 94% and 100%, and the split-half values were 98% and 92%, respectively, which indicated that an appropriate number of components were selected for each model.

### 3.2. Feature Extraction and Data Fusion

**Figure 2:**
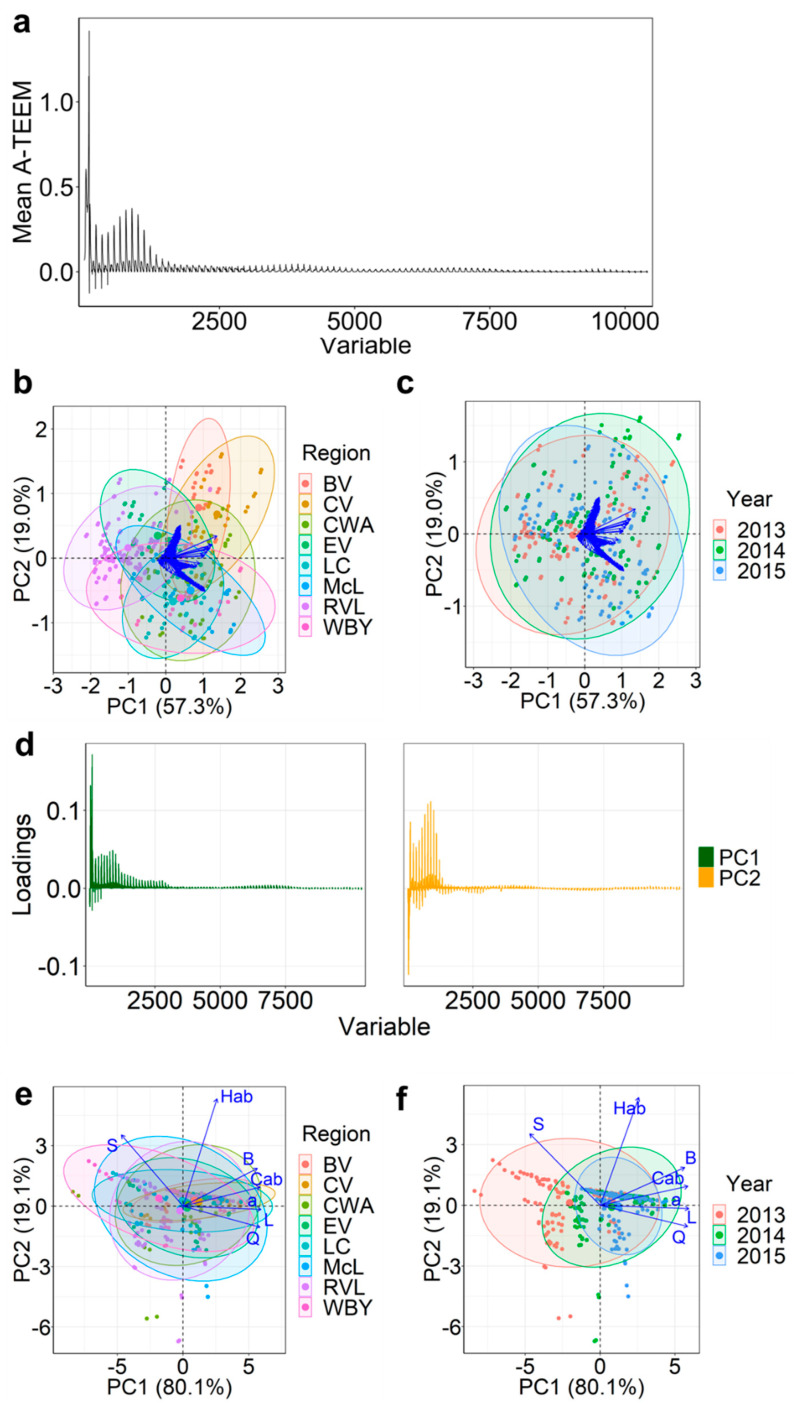
Absorbance-transmission and fluorescence excitation-emission matrix (A-TEEM) results showing (**a**) mean intensity of variable-level fused data, as well as PCA biplots showing scores and loadings on PC1 and PC2 with confidence ellipses for grape extract A-TEEM Train dataset (10,416 variables, blue arrows—labels not shown) according to (**b**) region and (**c**) year sampled, the A-TEEM variable loadings (**d**) on PC1 and PC2, and CIELAB Train dataset (7 variables, blue arrows) according to (**e**) region and (**f**) year sampled. Region abbreviations: BV, Barossa Valley; CV, Clare Valley; CWA, Coonawarra; EV, Eden Valley; LC, Langhorne Creek; McL, McLaren Vale; RVL, Riverland; WBY, Wrattonbully.

Prior to wine sensory score predictions, two datasets were prepared from grape extract spectra, which were each split into Train (85% of samples, *n* = 378) and Test (15% of samples, *n* = 66) arrays for the calibration and external validation of XGBoost regression models. This split ratio was chosen due to the number of XGBoost model features obtained from the A-TEEM dataset, with the number of features having been shown to influence the optimal ratio for data splitting [37]. The simplest dataset (Figure 2a) involved variable-level data fusion of scaled absorbance-transmission wavelengths from 240 to 700 nm (variables 1 to 93, with 5 nm increments) with scaled 2D EEMs (variables 94 to 10,416) to give the fused A-TEEM dataset. It is difficult to interpret 2D EEM spectra due to overlapping signals; however, the peaks are most likely related to the fluorophore groups identified using PARAFAC. The second dataset involved feature-level data fusion of A-TEEM data and CIELAB colour coordinates using principal component analysis (PCA). A-TEEM and CIELAB datasets were chosen because fluorescent fingerprints and colour matrices have been shown to contain valuable information for the prediction of sensory characteristics in studies specifically investigating wine sensory predictions [13,26,27] as well as other food products [8]. Furthermore, fluorescence and colour data can be readily obtained from an Aqualog spectrophotometer via a simple and quick procedure for spectral data acquisition.

The combination of multiple datasets has been achieved with the use of PCA for unsupervised dimension reduction and extraction of relevant features, yielding the principal components (PCs) [4]. Accordingly, PCA was conducted separately on A-TEEM and CIELAB Train datasets, and PCs that explained more than 1.0% of the variance were selected. Based on this approach, seven and two PCs were selected from A-TEEM and CIELAB data, respectively, after assessing the scree plot (Figure S2 of the Supplementary Material). Figure 2b,c show samples grouped by region and year on the first two PCs based on the A-TEEM data that cumulatively explained 76.3% of the variance between samples. A total of 92.8% of the variance was explained by the seven selected PCs (Figure S2a).

The A-TEEM variable loadings on PC1 and PC2 (Figure 2d) show the variables responsible for the largest discrimination of grape extract samples, with variable loadings for PC3-7 shown in Figure S3 of the Supplementary Material. The peaks and troughs could relate to the fluorophores identified in PARAFAC Models 1 and 2 (Figure 1). The ten highest A-TEEM variables contributing to PC1-7 are shown in Figure S4 of the Supplementary Material. Of relevance, absorbances in the UV region at 240 nm, 245 nm, and 270 to 280 nm, which relate to flavan-3-ol compounds in grape and wine [12], and in the visible region at 530 to 540 nm, which relate to anthocyanin (red-coloured) compounds [12], were among the most important contributors, and all were positively correlated to PC1 (Figure S5a of the Supplementary Material). The ten most important A-TEEM variables contributing to PC2 were visible wavelengths 525 to 550 nm from the absorbance-transmission data, and variables ‘772’ and ‘882’ to ‘884’ within the fluorescence region (Figure S4b). Wavelengths 525 to 550 nm were negatively correlated to PC2 (Figure S5a), and EEM variables ‘772’ and ‘882’ to ‘884’ were positively correlated (Figure S5b).

Figure 2b shows Barossa Valley (BV) and Clare Valley (CV) scores being slightly separated from Riverland (RVL) samples along PC1, indicating that RVL samples were lower in phenolic and anthocyanin compounds based on lower absorbance values at 240–280 nm and 540 nm, in accordance with previously reported A-TEEM results for wines [12]. Langhorne Creek (LC), McLaren Vale (McL), Coonawarra (CWA), and Wrattonbully (WBY) were positioned in the negative direction along PC2, being indicative of higher anthocyanin content compared with other regions based on wavelength variables. These regions were slightly separated from BV and CV, which were found in the negative direction of PC2, and more closely related to phenolic compounds (as projected using PARAFAC). PCA of A-TEEM data had difficulty separating samples by vintage (Figure 2c), indicating there were minor differences in the data associated with these grape extract spectral fingerprints. There appears to be multi-collinearity within the A-TEEM dataset according to PCA (Figure 2b,c), but that should not affect the performance of the XGBoost algorithm.

Two PCs accounted for 99.2% of the explained variance using PCA of the CIELAB dataset, with PC1 and PC2 explaining 80.1% and 19.1% of the variance, respectively, with grouping by region (Figure 2e) and vintage (Figure 2f). In contrast to the A-TEEM results, the biplot in Figure 2e indicates that PCA based on CIELAB parameters for grape extracts provided little separation according to the South Australian GI where the fruit was sampled. In terms of the vintage year, there was a slight separation along PC1, with 2013 samples tending to spread to the left along the axis. PC1 was related to colour components, C*ab (chroma), L*, a*, b*, and Q* in the positive direction, and less so to H*ab (hue angle), with S* (saturation) being negatively correlated. Thus, 2013 samples were deemed to be higher in saturation and were closer to the CIE green/blue colour space. Hue angle was the highest positive contributor to PC2, but there was minimal separation of region or year along this PC. Evidently, there appears to be a level of redundancy present in the dataset, although this is circumvented by extracting features from PCA and is not likely to influence the performance of prediction models.

A feature-level fused dataset with nine variables was constructed by extracting the sample scores on the seven PCs from PCA using A-TEEM data and two PCs from PCA using CIELAB data, based on the chemical information contained for marker variables that discriminated samples as discussed above. PARAFAC components were not put forward for constructing a feature-level data fused array as PCA of A-TEEM and CIELAB datasets required less computational time, which is an important consideration for developing a rapid analytical procedure. Nonetheless, if applying this proposed sensory modelling approach to other food matrices, other options for data pre-processing, level of data fusion, and feature selection could certainly be considered.

### 3.3. Performance of XGBoost Models

Predictive modelling of 22 wine sensory attribute scores [22,26,27,28] with an XGBoost algorithm was undertaken once datasets from variable-level and feature-level data fusion had been assembled from the grape extract A-TEEM spectra and CIELAB values. Notably, not all compounds responsible for aroma, flavour, taste, and mouthfeel characteristics necessarily fluoresce or absorb UV or visible light, or they may be in too low abundance to contribute greatly to a molecular fingerprint. Instead, marker variables within the A-TEEM fingerprint would be used for machine learning by the XGBoost algorithm. Figures S6–S27 (Supplementary Material) show the A-TEEM variable loadings on LVs determined by PLS, which was used as a dimension reduction step prior to XGBoost modelling, along with the gain of each LV, being a score that indicates the relative importance of a variable for building the boosted decision trees in the XGBoost algorithm. The gain of PCs obtained from PCA and used for XGBoost models with fused A-TEEM and CIELAB datasets are shown in Figures S28–S49 of the Supplementary Material. In the end, the ability to make robust predictions of sensory attributes can depend on the relationships between variables that are directly linkable (e.g., polymeric flavan-3-ols that elicit an astringency response or anthocyanins that impart red colour) or act as markers (i.e., components that relate in some way to those that may be directly linked to a sensory response) in the spectral fingerprints underpinning the modelling, or a combination thereof.

**Figure 3:**
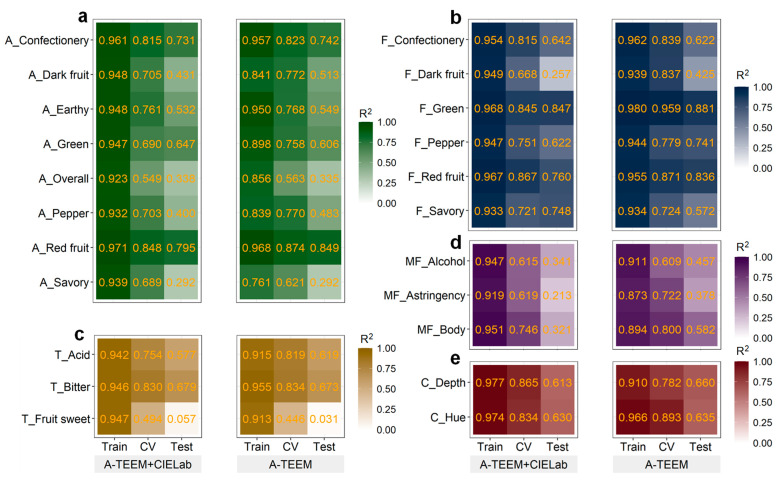
Summary of coefficient of determination (R^2^) values for XGBoost Train (calibration), cross-validation (CV), and Test (externally validated) models using feature-level (A-TEEM and CIELAB datasets) or variable-level (A-TEEM only) data fusion methods from the analysis of grape extracts for the prediction of wine attribute scores associated with (**a**) aroma, (**b**) flavour, (**c**) taste, (**d**) mouthfeel, and (**e**) colour.

**Figure 4:**
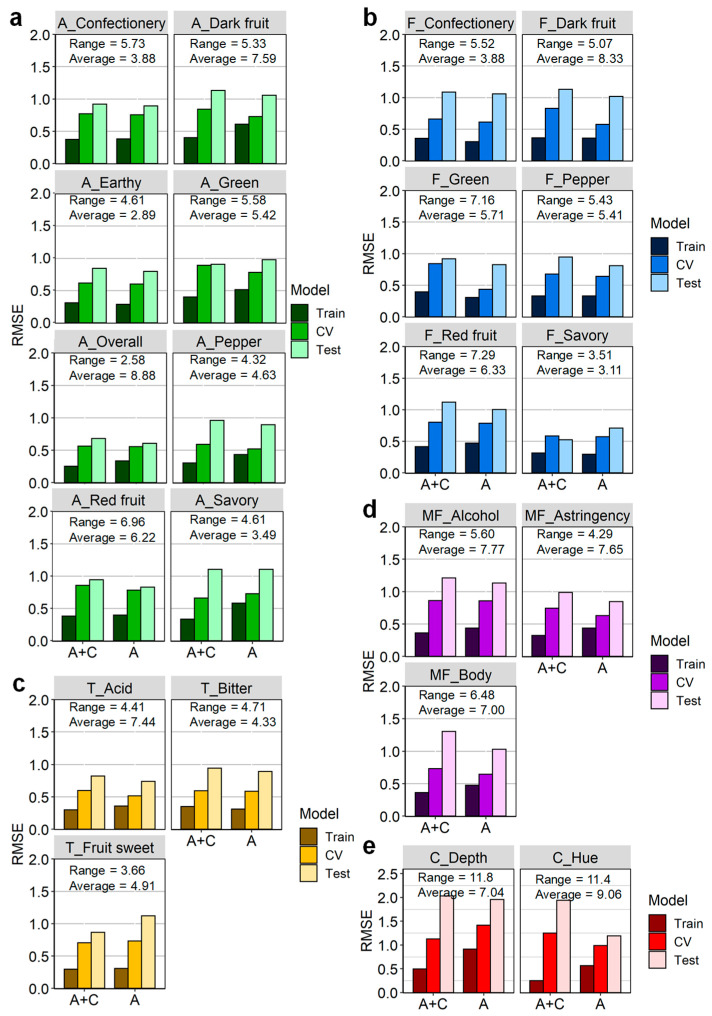
Bar charts of root mean square error (RMSE) values for XGBoost Train (calibration), cross-validation (CV), and Test (externally validated) models using feature-level (A + C, A-TEEM and CIELAB datasets) and variable-level (A, A-TEEM only) data fusion methods from analysis of grape extracts for the prediction of wine attribute scores related to (**a**) aroma, (**b**) flavour, (**c**) taste, (**d**) mouthfeel, and (**e**) colour. Range and average score values for Test datasets are displayed for each wine sensory attribute.

Figure 3 compares the coefficient of determination (R^2^) results of the two data fusion methods (feature and variable) for XGBoost Train, CV, and Test models for the predicted wine sensory attributes. The fusion methods are segregated, and Figure 3 is coloured according to the stimuli of the sensory attribute: aroma, flavour, taste, mouthfeel, and colour. Model accuracy based on RMSE was determined for predictions of each attribute, as summarised in Figure 4, which again is separated by the fusion method and coloured by sensory stimuli. The range and average score for each predicted sensory parameter of the Test datasets are displayed to aid the interpretation of RMSE results. Models were ultimately evaluated by examining the externally validated Test model R^2^ and RMSE values. It is apparent that both data fusion methods performed similarly; however, models with A-TEEM alone (variable-level data fusion) tended to have slightly better performance and more accurate predictions than models developed with feature-level fused A-TEEM and CIELAB (A-TEEM + CIELAB) datasets (Figure 3 and Figure 4). Furthermore, models were prone to overfitting, as seen with the R^2^ Train values being considerably higher than the R^2^ Test prediction models in some cases, which perhaps alludes to one of the limitations of decision-tree-based algorithms lacking the ability to generalise to new variations.

### 3.4. Prediction of Sensory Scores

Using the variable-level fused data, externally validated XGBoost predictive models for confectionery, dark fruit, earthy, pepper, and red fruit aroma attributes yielded R^2^ values of 0.742, 0.513, 0.549, 0.483, and 0.849, respectively, which were higher than the models using feature-level data fusion (Figure 3a). Yet, aroma attribute score RMSE values for the Train, CV, and Test models using either fusion method were similar (Figure 4a), indicating that there were small differences in model accuracy. For green and overall intensity aroma scores, XGBoost coupled with fused A-TEEM and CIELAB datasets had higher Test R^2^ values of 0.647 and 0.338, respectively. The Test R^2^ value (0.292) for savoury aroma prediction was equal for both models.

Figure 3b and Figure 4b summarise the performance of XGBoost regression models for the prediction of wine flavour attribute scores for dark fruit, red fruit, confectionery, green, pepper, and savoury. Encouragingly, externally validated models using only A-TEEM data had R^2^ values of 0.881, 0.741, and 0.836 for green, pepper, and red fruit flavour predictions, respectively, which were higher than models using fused A-TEEM and CIELAB data (R^2^ = 0.847, 0.622, 0.760, respectively). Large differences in model performance were observed for savoury and dark fruit flavour Test model predictions using variable-level fused data (A-TEEM) with corresponding R^2^ values of 0.425 and 0.572, and models using feature-level fused data having R^2^ values of 0.257 and 0.748, with differences also observed in RMSE values (Figure 4b).

Human perception of aroma and flavour through olfaction is due to groups of volatile compounds such as monoterpenoids, esters, and methoxypyrazines, to name a few, which are present in trace concentrations. These compounds potentially do not fluoresce or absorb UV-Vis wavelengths with enough intensity or at distinctive enough wavelengths to contribute to the spectral fingerprint. Whether they do or do not, this highlights the advantage of the chemometric and machine learning approach with A-TEEM spectral data, whereby the molecular fingerprints are processed and employed by the XGBoost algorithm for the predictions. By comparing prediction models of aroma and flavour attribute scores with higher and lower performance using A-TEEM only, such as red fruit and dark fruit or green and savoury, there appears to be no distinct pattern in variable loadings that distinguish a high or low performing aroma or flavour attribute prediction model (Figures S6–S19). This is the same scenario for predictions made with the fusion of A-TEEM and CIELAB, where there is no common trend in PCs for higher or lower performing models (Figures S28–S41). In general, it appears that absorbance wavelengths highly contributed to LVs and PCs that had high gain for XGBoost modelling. In contrast, fluorophore chemical information (EEM variables) had moderate contributions, but the entire spectra were still used, especially for the prediction of green flavour.

Test model performance for taste attribute predictions (Figure 3c and Figure 4c) exhibits a slightly better R^2^ value of 0.679 for bitter taste using PCs of A-TEEM and CIELAB datasets, whereas, for acid taste prediction, models using A-TEEM alone performed better with an R^2^ value of 0.619. The corresponding RMSE values for externally validated models were below 1.0 using either data fusion method, although variable-level data fusion models (A-TEEM data) were more accurate by 5.3% and 10.2% for bitterness and acidity, respectively. Interestingly, compounds that impart bitter taste were tentatively assigned to PARAFAC Model 1 components (monomeric catechins) [24], with this information embedded in the spectral data and likely used by the XGBoost algorithm to make these predictions (Figures S21 and S43 of the Supplementary Material). Both models had difficulty predicting fruit sweetness taste for the Test dataset, with R^2^ values below 0.06. This may be due to a lack of a spectral signature that related well to this specific wine sensory attribute (see Figures S22 and S44) and accords with the previous study that employed mid-infrared spectroscopy of grape extracts for the prediction of wine sensory attribute scores [28].

XGBoost Test prediction models for mouthfeel attributes (trigeminal and textural sensations associated with alcohol intensity, astringency, and body) using A-TEEM alone had R^2^ values of 0.457, 0.378, and 0.582, respectively (Figure 3d). These values are higher than models using fused A-TEEM and CIELAB data, having R^2^ values of 0.341, 0.213, and 0.321. RMSE values for A-TEEM models were accordingly lower by 0.079–0.271, with body score predictions having the largest difference between the two data fusion models (Figure 4d). XGBoost model performance was surprisingly low for the astringency score, considering that polyphenolic compounds potentially stimulating an astringent mouthfeel in wine [24] were elucidated using PARAFAC of grape extract EEMs. Furthermore, absorbance-transmission data from wavelengths in the range 240–280 nm should correspond at least in part to the presence of the same suite of polyphenolic compounds that impart astringent mouthfeel, and these wavelengths were the highest contributors to PC1 according to PCA of A-TEEM data (Figure 2b,c). This highlights the non-linear relationship between grape chemical composition and this wine mouthfeel attribute and reflects the complexity of a sensory trait such as astringency, which can be influenced by multifactorial interactions among wine matrix components [38].

CIELAB values are commonly used to assess the colour parameters of wine and other food products [4], but wine colour attribute predictions were not significantly improved by including CIELAB values with A-TEEM when compared to Test models using A-TEEM alone (Figure 3e and Figure 4e). This is perhaps not so surprising, considering that measures of colour are embedded within the A-TEEM spectra. Hue and colour depth score predictions for the externally validated A-TEEM models had slightly higher R^2^ values of 0.635 and 0.660 than the feature-level fused model. The RMSE results for each colour attribute prediction were lower for A-TEEM only models, with hue RMSE considerably lower by 38.5%.

In the present study, which used stored grape samples and wine sensory data from previous work [22,26,27,28], data from all vintages were included together for each sensory attribute prediction, with the XGBoost algorithm performing encouragingly well. For example, using only A-TEEM variables, Test models for confectionery and red fruit aroma, and green, pepper, and red fruit flavour had R^2^ values ranging between 0.741–0.881 and RMSE values of 0.891, 0.782, 0.825, 0.810, and 1.00, respectively. The RMSE values for these models were 11–16% of the range for the corresponding sensory attribute score in the Test dataset, which is a highly promising outcome for a single “sensor”. Additional regression models in this study gave a percentage of RMSE of the range being ≤16.0%; these were body, mouthfeel, and hue. All other models using A-TEEM data had RMSE% of the range between 16.5% and 24.0% when the fruit sweetness taste model was removed, which itself had an RMSE equivalent to 30.6% of the score range. In contrast, previous research made predictions based on data from separate years of vintage due to high levels of variability between 2013, 2014, and 2015; in that case, achieving R^2^ values of less than 0.6 for CV models of all wine attribute score predictions using PLS with mid-infrared spectroscopy [28]. In other studies, a suite of grape extract chemical measurements, including those from GC-MS and HPLC instrumentation, were used to make predictions of wine sensory traits that were normalised based on vintage [26,27], which was not the case in the current study. Considering this, it is important to carefully assess the modelling objective and apply the appropriate pre-processing procedure for future studies. Although that previous work achieved similar model performance to the current study, comparisons are difficult because the former models were not validated with an external dataset.

There are challenges that remain with predicting food sensory attributes from raw material chemical constituents. Model accuracy for sensory attributes that lack a specific compound stimulus was shown to be lower, despite some other sensory attributes being predicted well. This may be because sensory attributes can be a construct involving multiple sensory modalities, such that the variability in descriptive analysis data from panellists may have been high. For other foodstuffs, the same suite of descriptors might be a result of different chemical compositions and may require new modelling. Even though a broad range of descriptors was demonstrated in this study and could be applied to food types with similar composition, for example, fruit jams or tea, future work is needed to investigate the applicability of the method to predict sensory attributes of other food products.

## 4. Conclusions

Machine learning modelling of grape extract spectral data with XGBoost regression proved to be a powerful and rapid analytical tool to assess wine sensory attributes from the raw material. XGBoost Test models using either variable- or feature-level data fusion to predict wine sensory attributes from five sensory modalities achieved respective R^2^ values above 0.5 for 15 and 13 out of the 22 sensory attributes analysed. The outcome was encouraging, considering the complexity involved in predicting sensory attribute scores of a biotransformed product from the starting material, in this case, using grape extract spectral data and wine sensory profiles to exemplify the approach. Overall, this technique can be proposed as an innovative and rapid method for the simultaneous prediction of food/beverage sensory characteristics from raw materials with a single spectrophotometer, which could be considered for further research on other food products and developed for routine testing to negate the need for employing and training a human sensory panel.

## Data Availability

The data presented in this study are available in the article and supplementary material.

## Supplementary Materials

The following supporting information can be downloaded at: https://www.mdpi.com/article/10.3390/foods12040757/s1, Figure S1: Map showing eight South Australian GIs where grapes were sampled from; Table S1: Values used in a grid-search to optimise XGB hyperparameters; Figures S2–S5: Supporting figures for PCA of A-TEEM and CIELAB datasets; Figures S6–S27: A-TEEM variable loadings used by XGBoost for the prediction of sensory attributes and the gain of each feature; Figures S28–S49: Gain of each PC from PCA of A-TEEM and CIELAB datasets used to predict sensory attributes.
