# Supplementary material for: Use of Machine Learning with Fused Spectral Data for Prediction of Product Sensory Characteristics: The Case of Grape to Wine
Source: Foods. 2023 Feb 9;12(4):757. doi: 10.3390/foods12040757 (PMC9955574; doi:10.3390/foods12040757)
Supplement: Supplementary file 1 [file foods-12-00757-s001.zip › foods-2129388-SI.pdf]

SUPPLEMENTARY MATERIAL FOR

Use of Machine Learning with Fused Spectral Data for Prediction of Product Sensory Characteristics: The Case of Grape to Wine

Claire E. J. Armstrong [1,2], Jun Niimi [2,3,†], Paul K. Boss [1,3], Vinay Pagay [1,2], David W. Jeffery [1,2,*]

[1] Australian Research Council Training Centre for Innovative Wine Production, The University of Adelaide, PMB 1, Glen Osmond, SA 5064, Australia

[2] School of Agriculture, Food and Wine, and Waite Research Institute, The University of Adelaide, PMB 1, Glen Osmond, SA 5064, Australia

[3] CSIRO Agriculture and Food, Locked Bag 2, Glen Osmond, SA 5064, Australia

* Correspondence: david.jeffery@adelaide.edu.au

[†] Present address: Division of Bioeconomy and Health, RISE Research Institutes of Sweden, Gothenburg, 412 76, Sweden

Table of Contents

	Page
**Figure S1.** Map of Australia with South Australia highlighted in red. Inset shows the eight South Australian GIs (and their abbreviations) where grape parcels were obtained in 2013, 2014, and 2015 (n = 74 in total).	**S2**
**Table S1.** Values used in grid-search to optimise XGB hyperparameters for the predictions of wine sensory attributes using A-TEEM data alone or feature-level fused A-TEEM and CIELAB data.	**S2**
**Figure S2:** Scree plot of the percentage of explained variance of principal components (PCs) from principal component analysis using (a) A-TEEM data, and (b) CIELAB colour coordinates	**S3**
**Figure S3.** A-TEEM variable loadings on PC3 to 7 from principal component analysis	**S3**
**Figure S4.** Percentage of contributions of the top ten highest contributing variables on (a) PC1 to (g) PC7, from principal component analysis (PCA) of A-TEEM data.	**S4**
**Figure S5.** Correlation matrix plot for the relationship between the seven selected principal components from principal component analysis (PCA) using A-TEEM data and the top ten most contributing variables	**S5**
**Figures S6-27. (a)** A-TEEM variable loadings on partial least squares (PLS) regression latent variables (LVs) and (b) gain brought by LVs for branches in the XGB algorithm.	**S6–S16**
**Figures S28-49.** The gain brought by principal components (PCs) of A-TEEM and CIELAB datasets after principal component analysis for branches in the XGB algorithm.	**S17–S22**

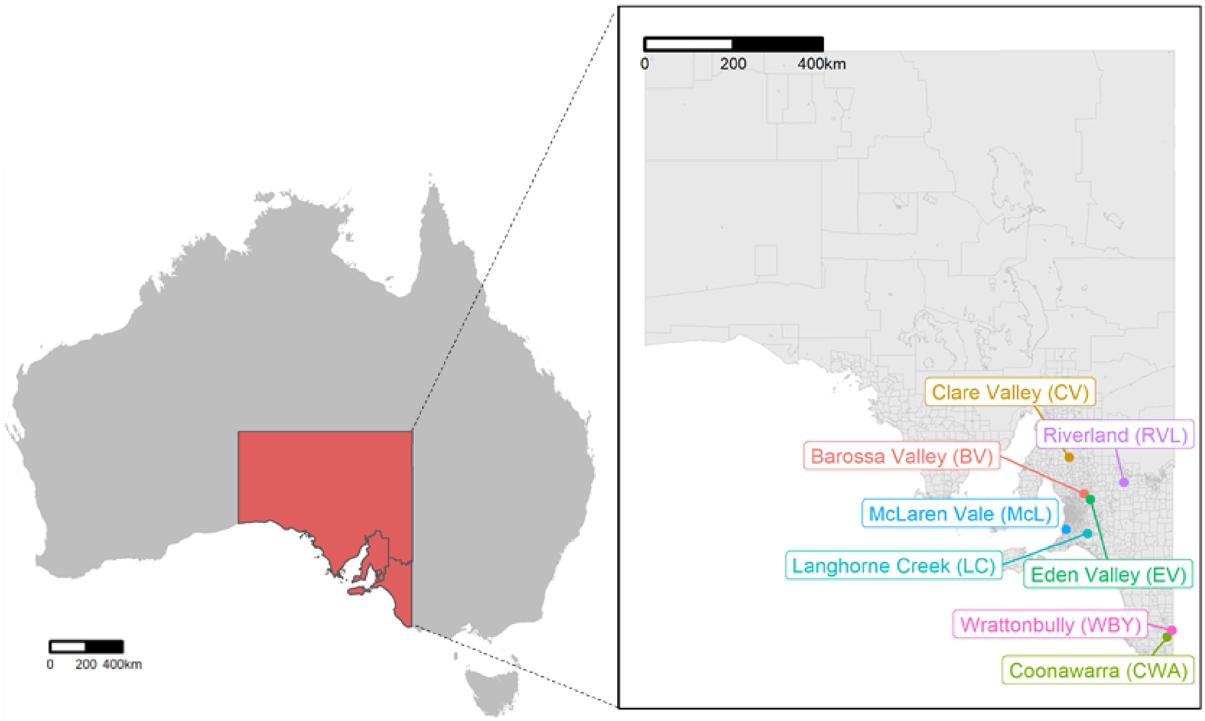

Figure S1. Map of Australia with South Australia highlighted in red. Inset shows the eight South Australian GIs (and their abbreviations) where grape parcels were obtained in 2013, 2014, and 2015 (n = 74 in total).

Table S1. Values used in grid-search to optimise XGB hyperparameters for the predictions of wine sensory attributes using A-TEEM data alone or feature-level fused A-TEEM and CIELAB data.

	Description	Grid search values for models using A-TEEM data	Grid search values for models using fused A-TEEM and CIELAB data
max_depth	Maximum depth of a tree (i.e. number of branches)	1, 2, 3, 4, 5, 6	1, 2, 3, 4, 5, 6
num_round	Maximum number of decision trees in ensemble	50, 100, 200, 300, 400, 500, 600	50, 100, 200, 300, 400, 500, 600
eta	Shrinkage value for feature weights to make boosting process more conservative	0.02, 0.04, 0.08, 0.1, 0.2, 0.3, 0.4, 0.5	0.02, 0.04, 0.08, 0.1, 0.2, 0.3, 0.4, 0.5
alpha	L1 regularization term on weights	1	1
lambda	L2 regularization term on weights	2	2
gamma	Before further partition on a leaf node of a tree, the minimum loss reduction (gamma) must be achieved.	0.2	0.4
compression	Method used for dimension reduction	PLS with 2 to 6 LVs	None
declutter	Method to remove clutter covariance on X-Block	GLSW (α = 0.02) or EPO (3 LVs)	GLSW (α = 0.02) or EPO (3 LVs)

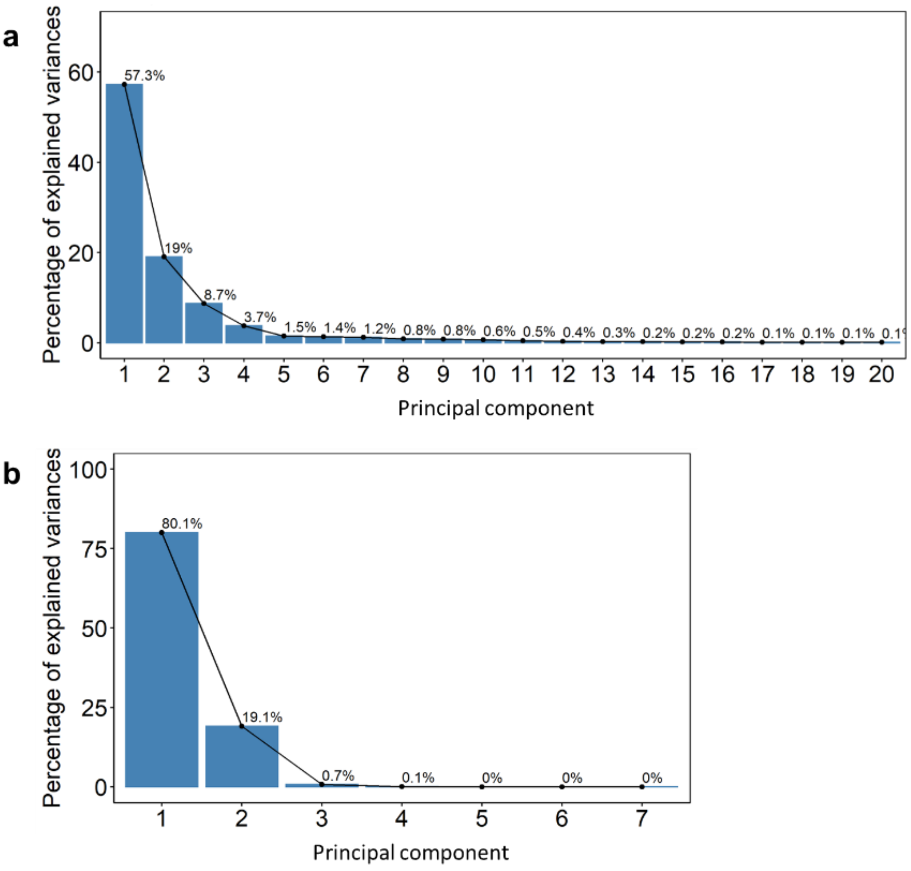

Figure S2. Scree plot of the percentage of explained variance of principal components (PCs) from principal component analysis (PCA) using (a) A-TEEM data, and (b) CIELAB colour coordinates.

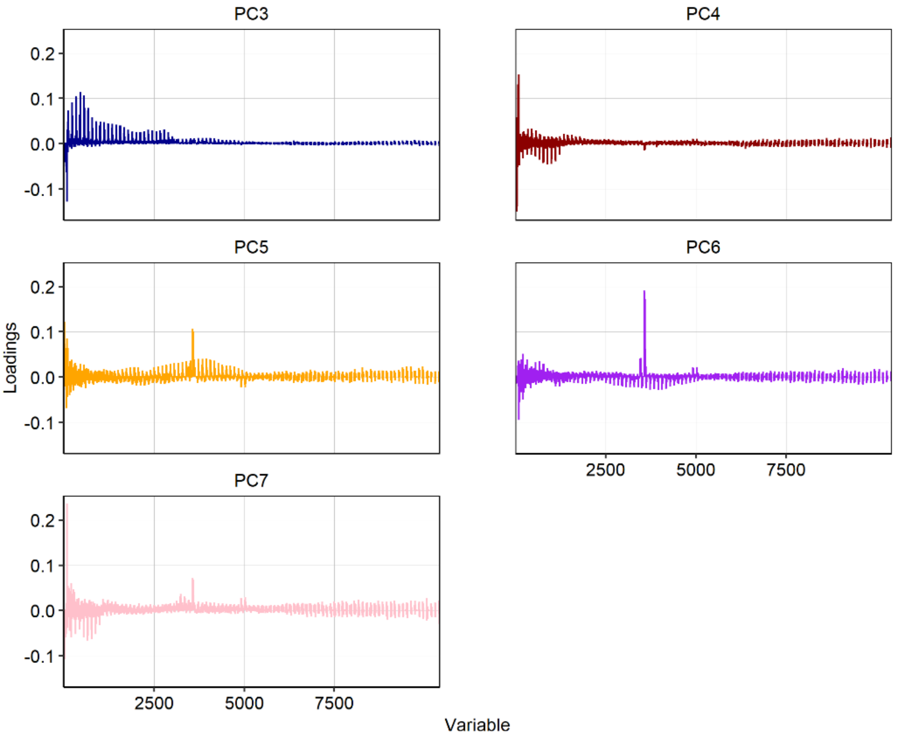

Figure S3. A-TEEM variable loadings on PC3 to 7 from PCA performed before feature-level data fusion.

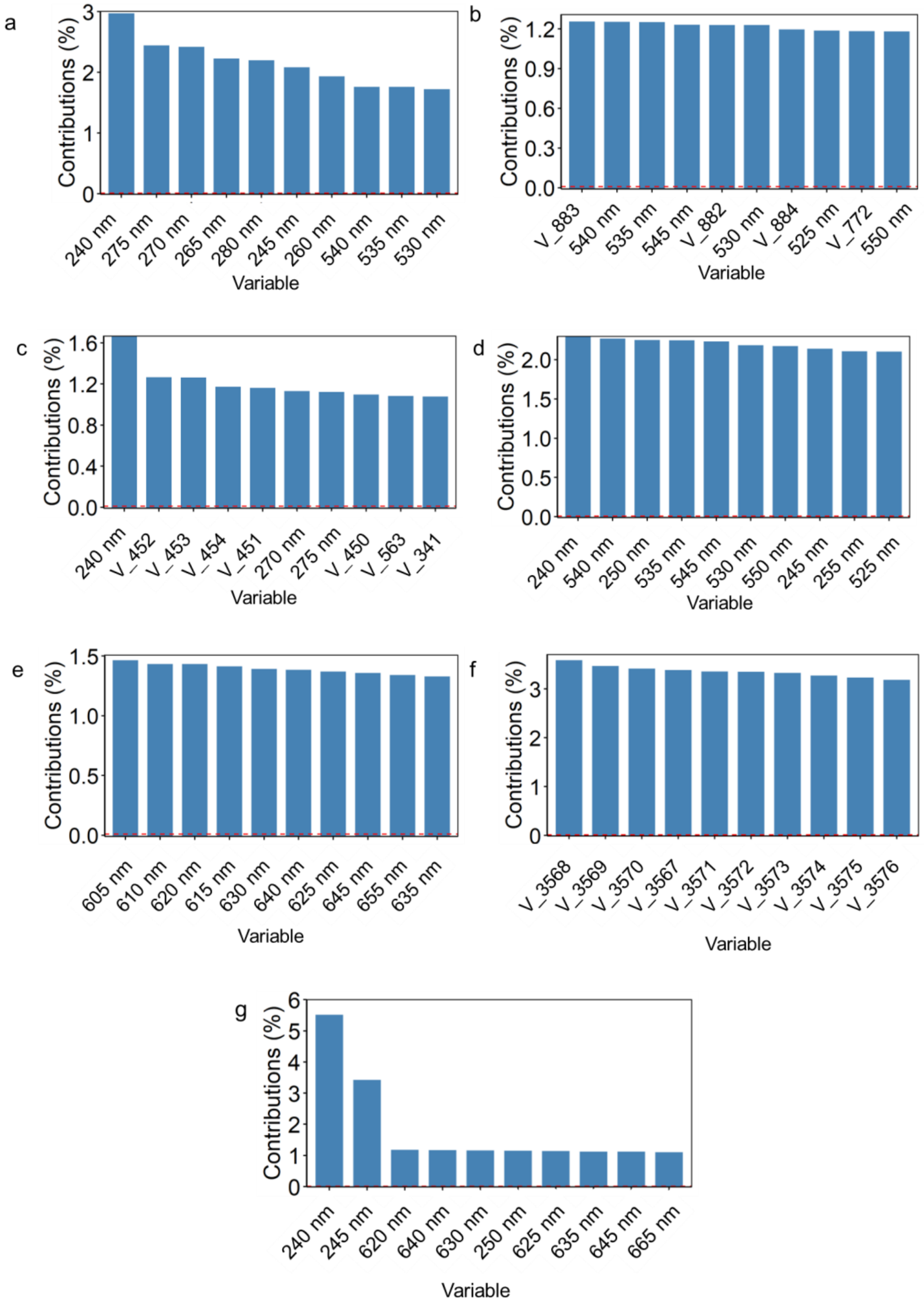

Figure S4. Percentage of contributions of the top ten highest contributing variables on (a) PC1 to (g) PC7, from principal component analysis of A-TEEM data.

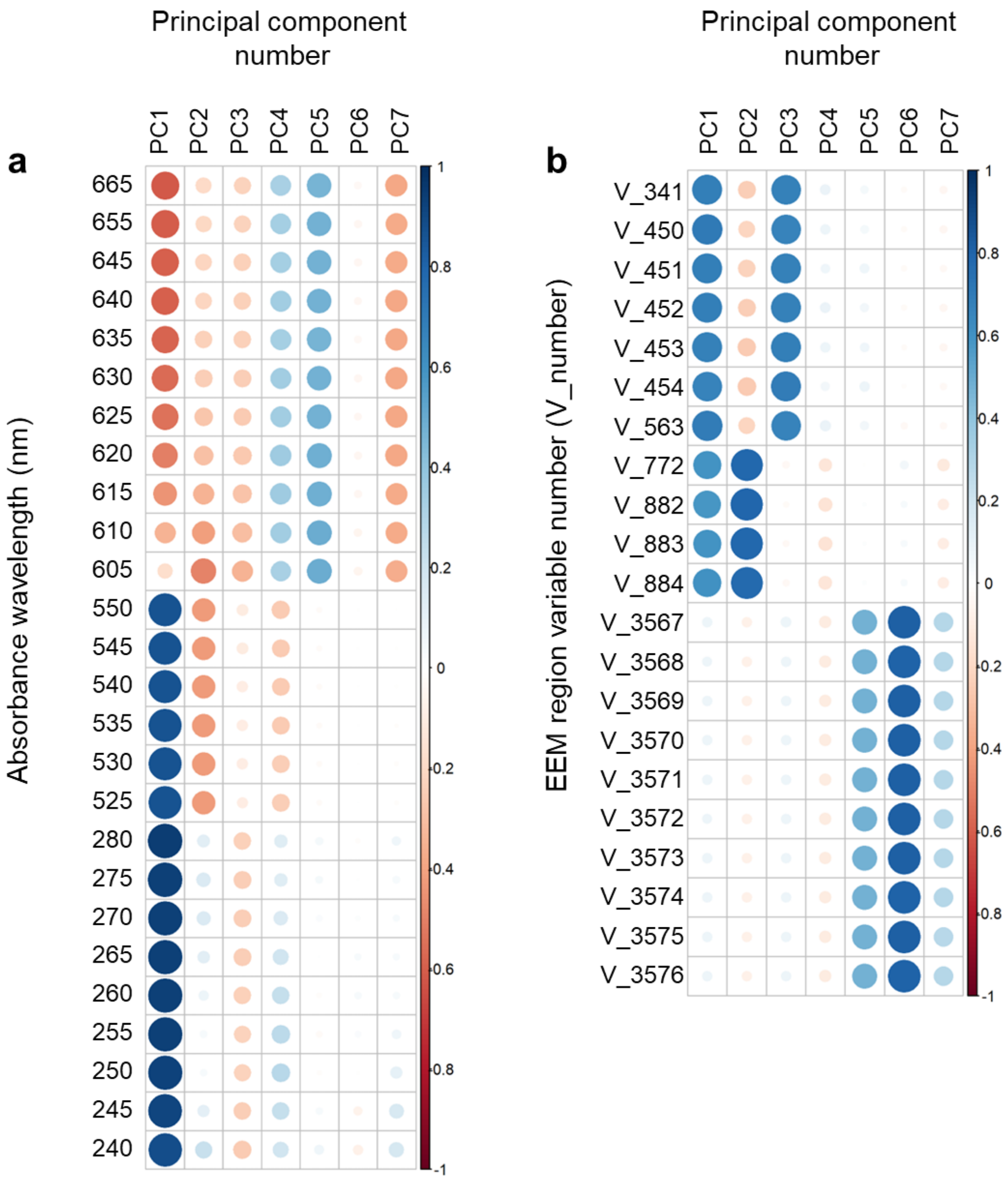

Figure S5. Correlation matrix plot for the relationship between the seven selected principal components (PCs) from principal component analysis using A-TEEM data and the top ten most contributing variables (see Figure S1) to the seven PCs, divided into (a) the absorbance region of A-TEEM, which is variable 1 to 93 and relates to absorbance wavelength 700 to 240 nm (with 5 nm increments), respectively, and (b) the 2D EEM region of A-TEEM, which is variable 94 to 10416. A strong positive linear correlation with a value of 1 between two variables is coloured dark blue and a strong negative linear correlation with a value of -1 is coloured dark red. The size of circles also depicts the strength of the relationship.

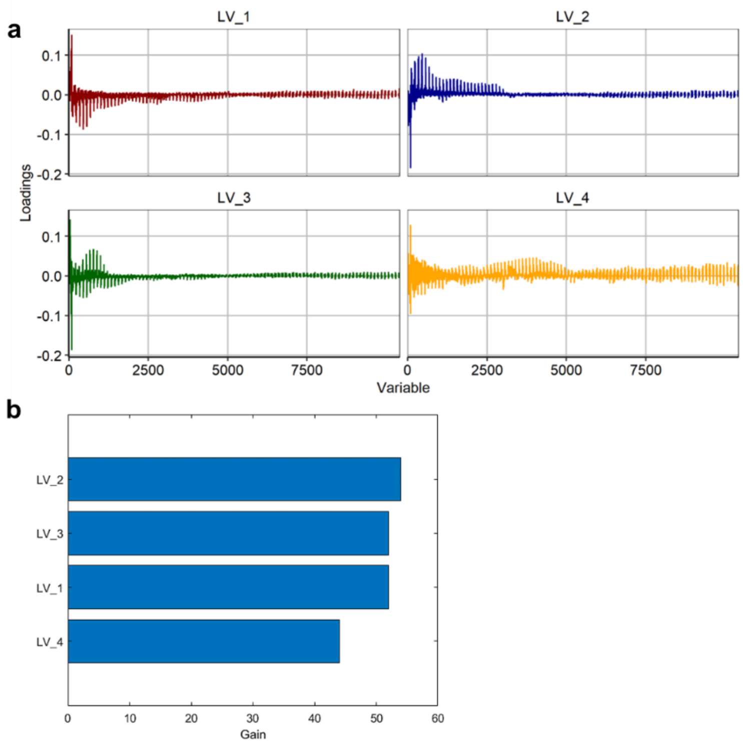

Figure S6. (a) A-TEEM variable loadings on partial least squares (PLS) regression latent variables (LVs) 1-4 used in the XGB algorithm to predict confectionery aroma attribute scores and (b) the gain of LVs.

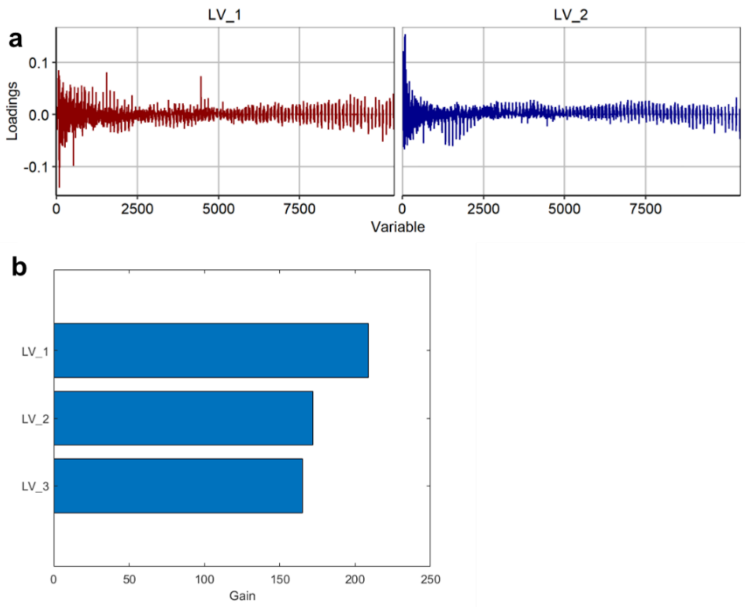

Figure S7. (a) A-TEEM variable loadings on PLS LVs 1 and 2 used in the XGB algorithm to predict dark fruit aroma attribute scores and (b) the gain of LVs.

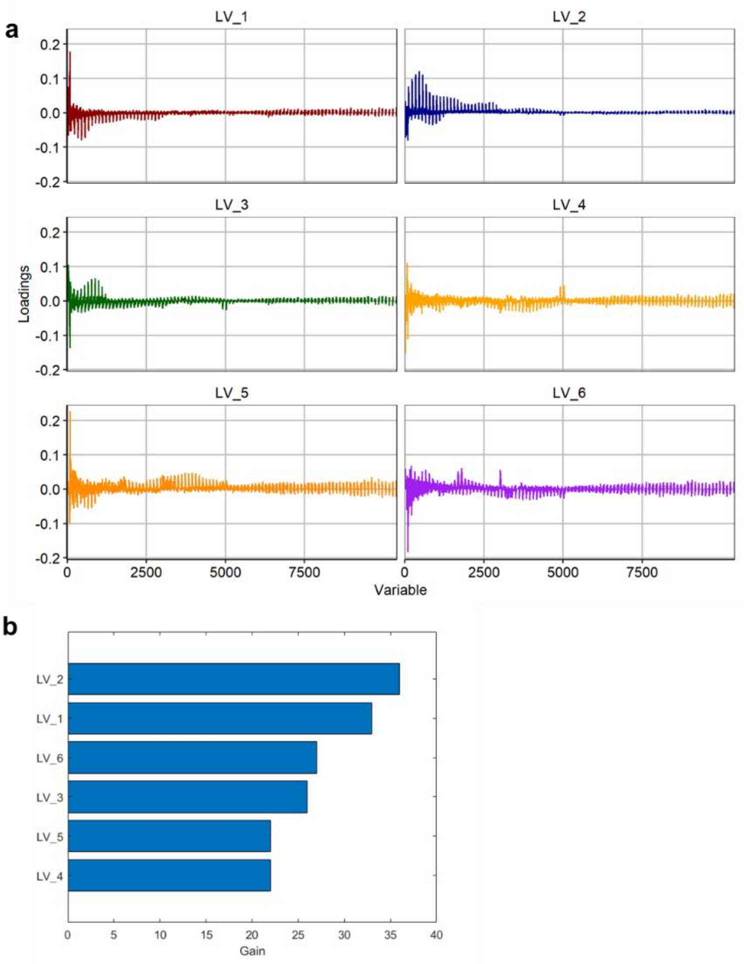

Figure S8. (a) A-TEEM variable loadings on partial least squares (PLS) regression latent variables (LVs) 1-6 used in our XGB algorithm to predict earthy aroma attribute scores and (b) the gain of LVs.

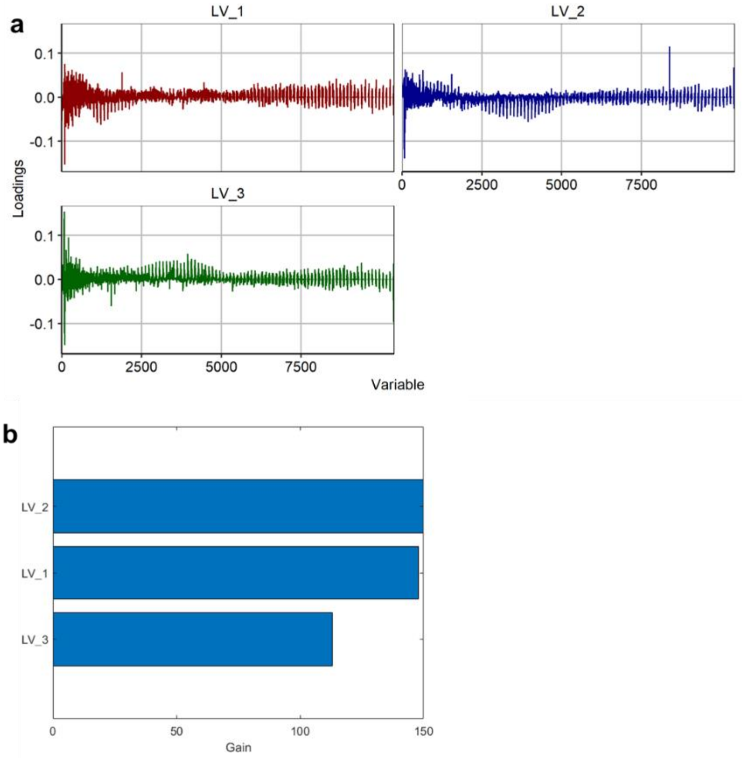

Figure S9. (a) A-TEEM variable loadings on PLS LVs 1-3 used in the XGB algorithm to predict green aroma attribute scores and (b) the gain of LVs.

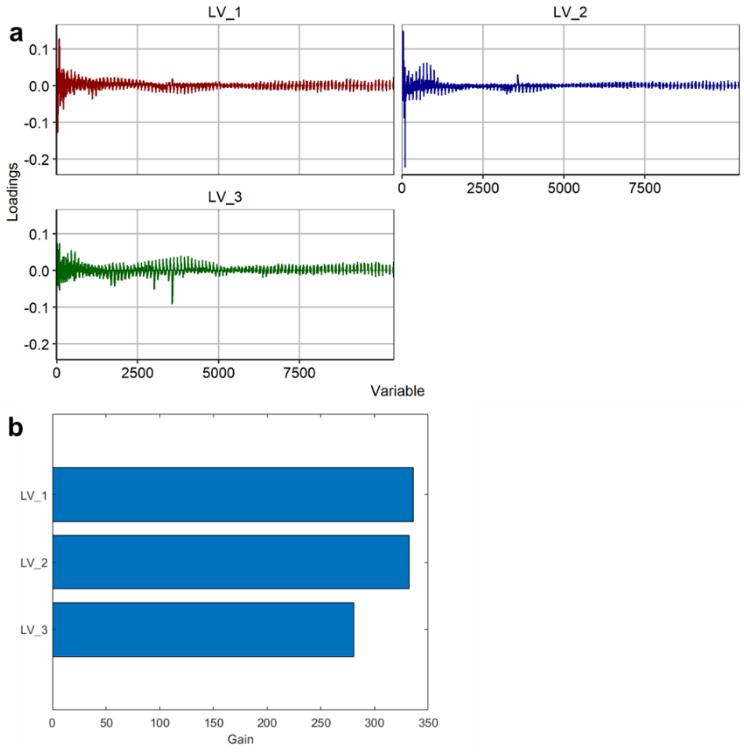

Figure S10. (a) A-TEEM variable loadings on partial least squares (PLS) regression latent variables (LVs) 1-4 used in the XGB algorithm to predict overall intensity aroma attribute scores and (b) the gain of LVs.

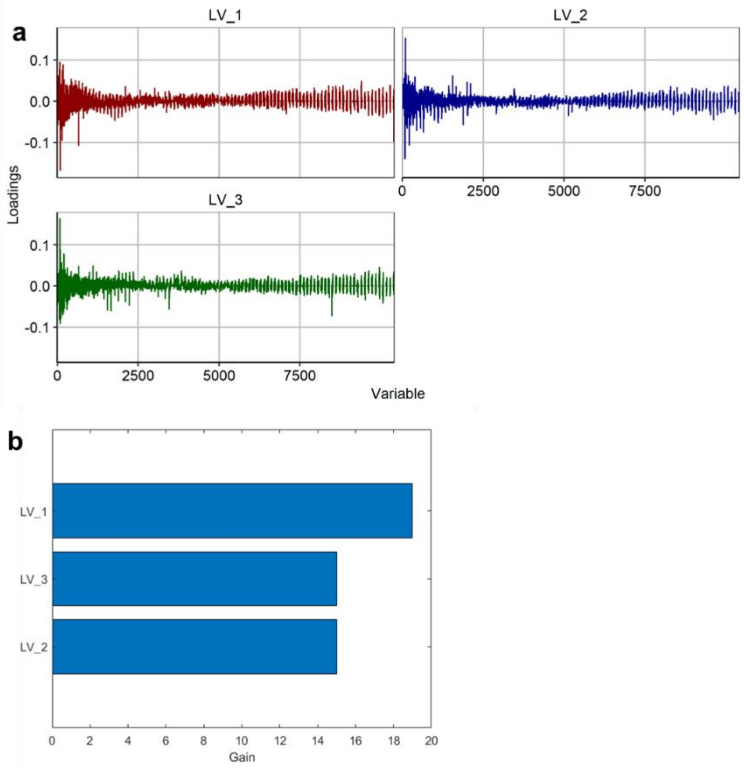

Figure S11. (a) A-TEEM variable loadings on PLS LVs 1-4 used in the XGB algorithm to predict pepper aroma attribute scores and (b) the gain of LVs.

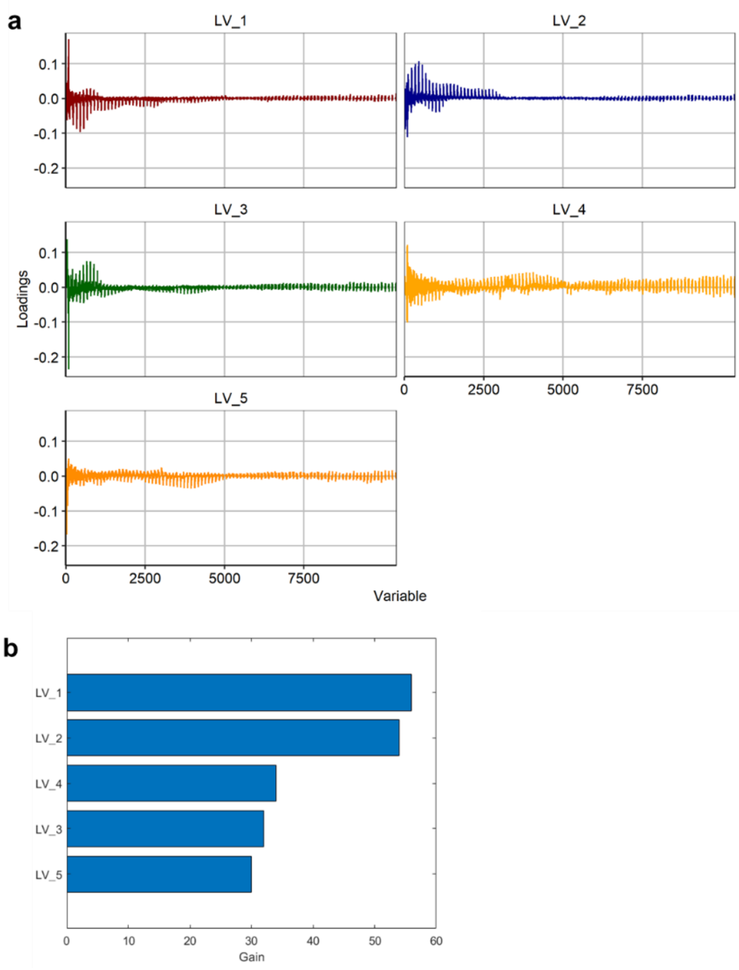

Figure S12. (a) A-TEEM variable loadings on partial least squares (PLS) regression latent variables (LVs) 1-4 used in the XGB algorithm to predict red fruit aroma attribute scores and (b) the gain of LVs.

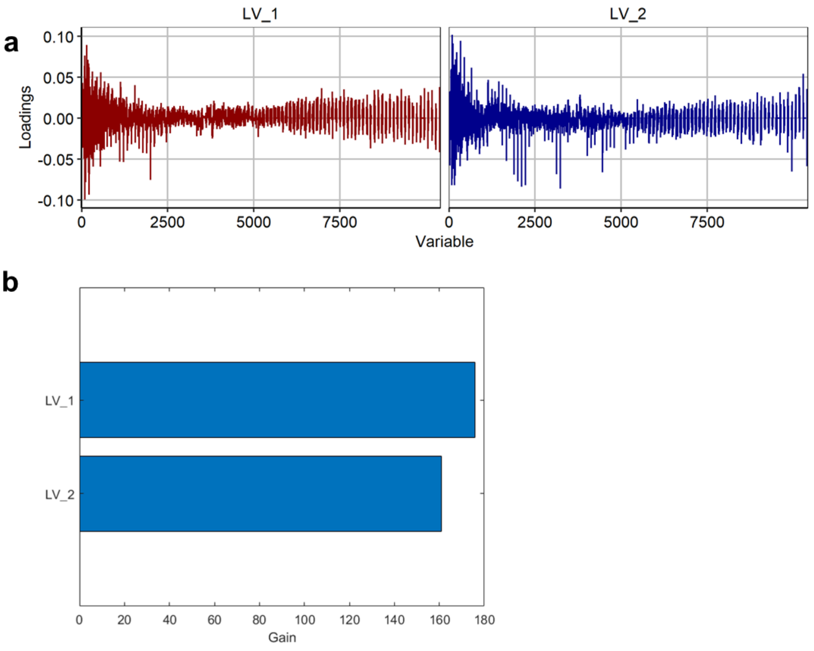

Figure S13. (a) A-TEEM variable loadings on PLS LVs 1-4 used in the XGB algorithm to predict savoury aroma attribute scores and (b) the gain of LVs.

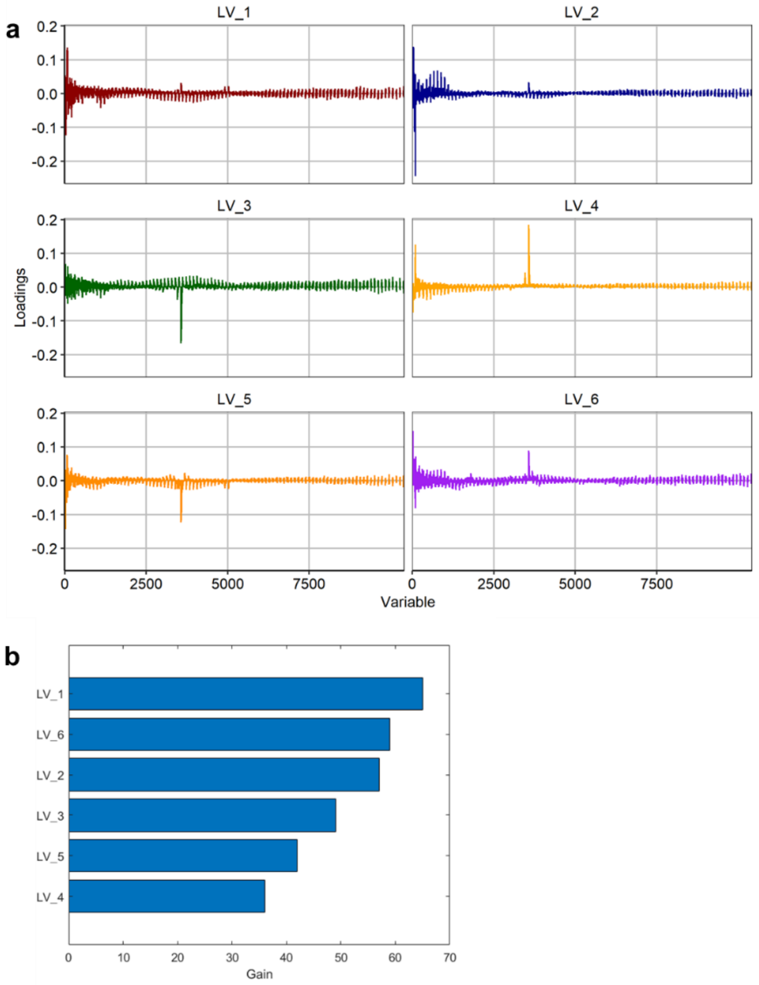

Figure S14. (a) A-TEEM variable loadings on partial least squares (PLS) regression latent variables (LVs) 1-4 used in the XGB algorithm to predict confectionery flavour attribute scores and (b) the gain of LVs.

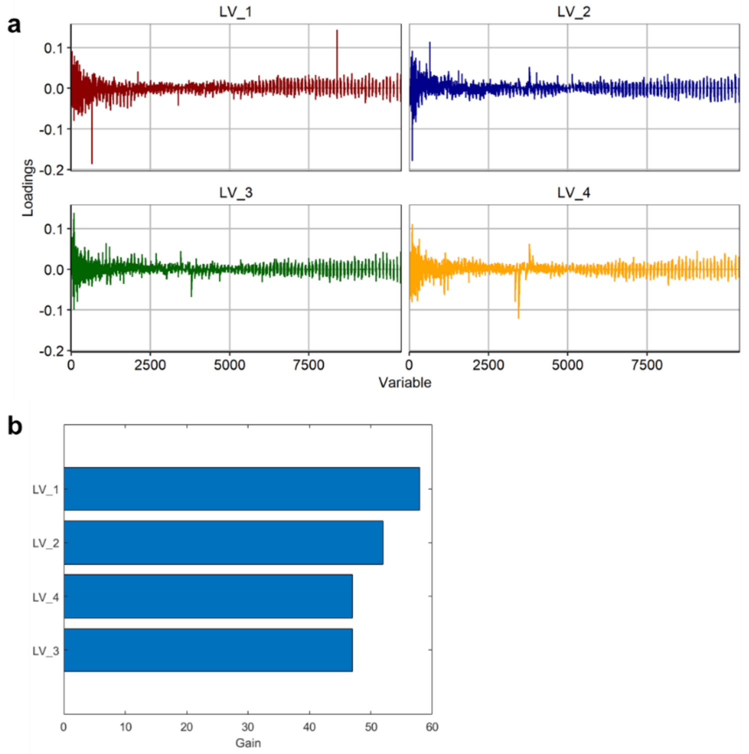

Figure S15. (a) A-TEEM variable loadings on PLS LVs 1-4 used in the XGB algorithm to predict dark fruit flavour attribute scores and (b) the gain of LVs.

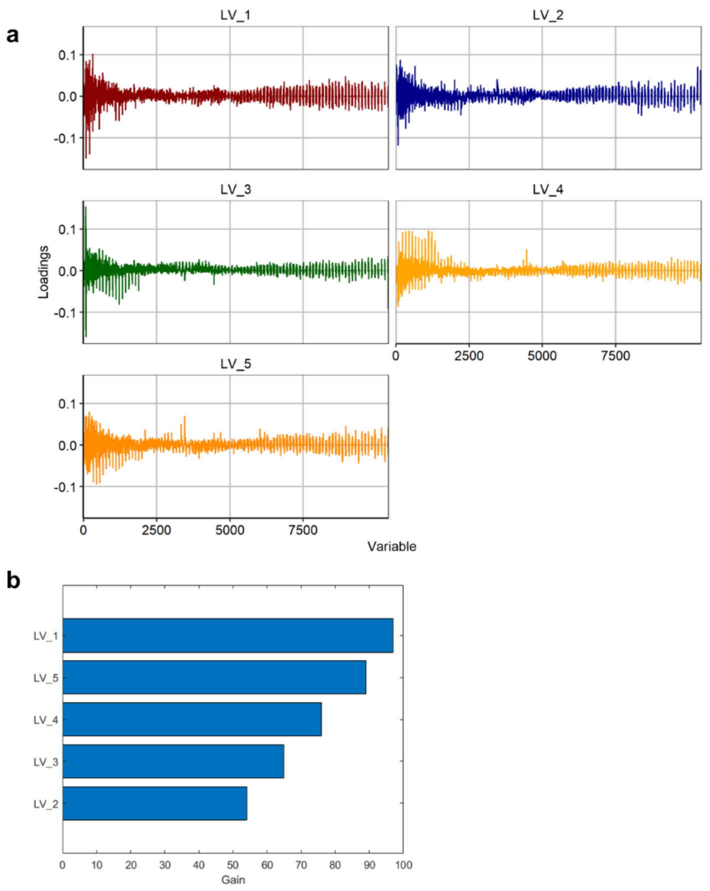

Figure S16. (a) A-TEEM variable loadings on partial least squares (PLS) regression latent variables (LVs) 1-4 used in the XGB algorithm to predict green flavour attribute scores and (b) the gain of LVs.

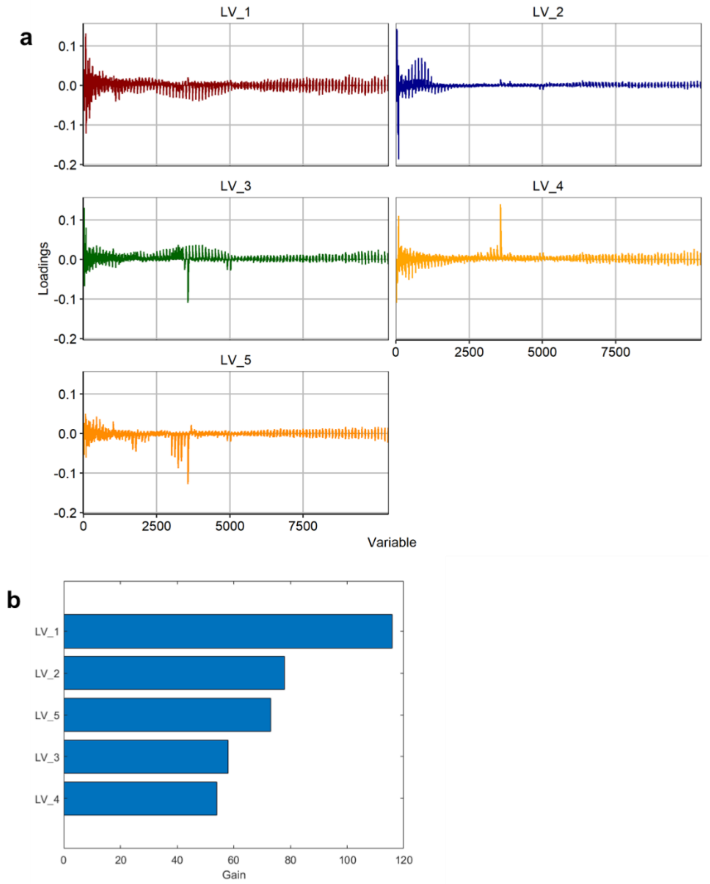

Figure S17. (a) A-TEEM variable loadings on PLS LV1-4 used in the XGB algorithm to predict pepper flavour attribute scores and (b) the gain of LVs.

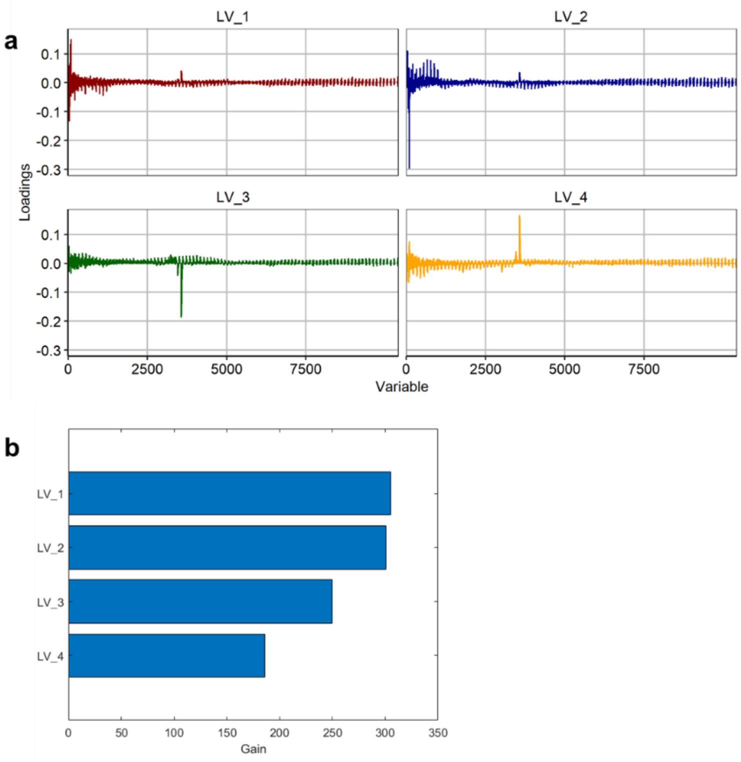

Figure S18. (a) A-TEEM variable loadings on partial least squares (PLS) regression latent variables (LVs) 1-4 used in the XGB algorithm to predict red fruit flavour attribute scores and (b) the gain of LVs.

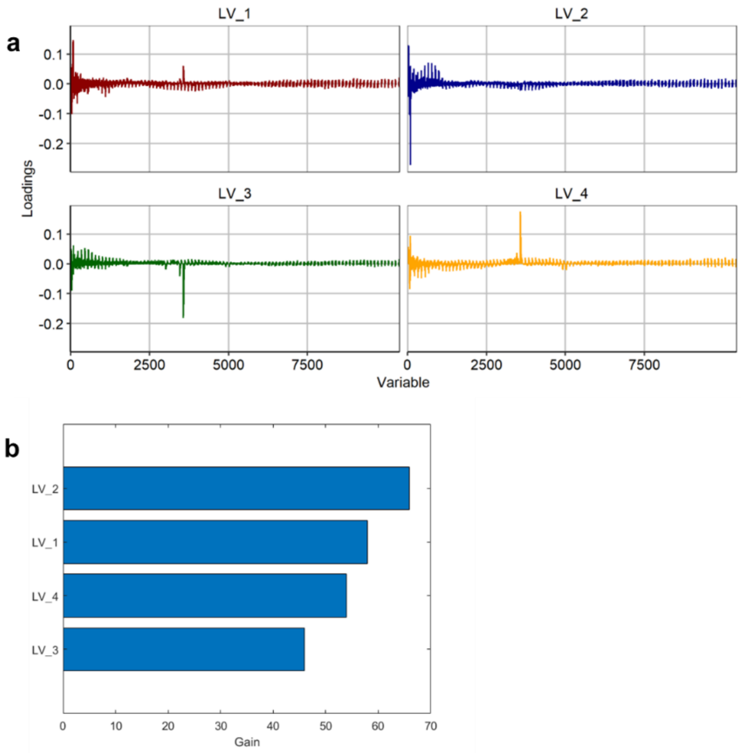

Figure S19. (a) A-TEEM variable loadings on PLS LVs 1-4 used in the XGB algorithm to predict savoury flavour attribute scores and (b) the gain of LVs.

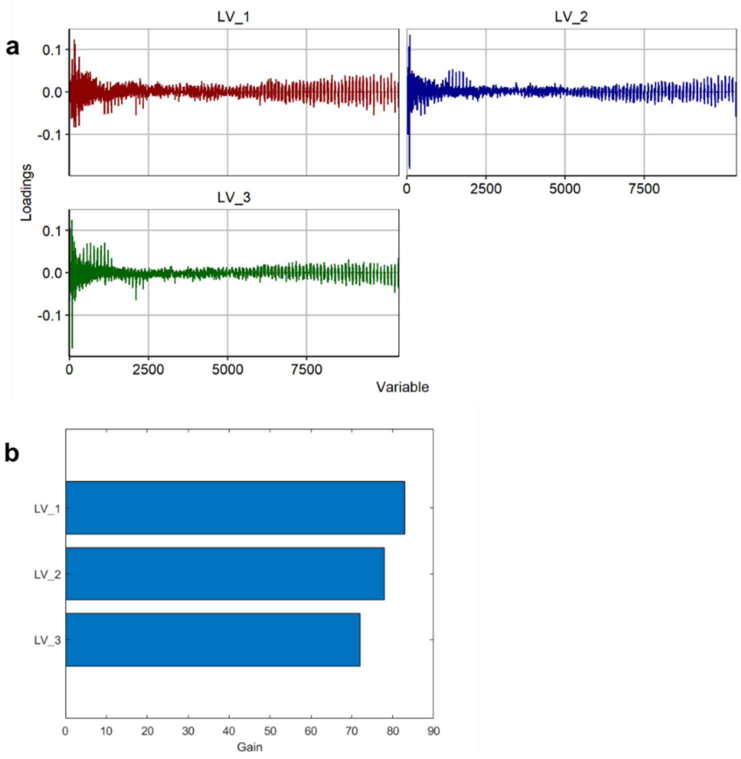

Figure S20. (a) A-TEEM variable loadings on partial least squares (PLS) regression latent variables (LVs) 1-4 used in the XGB algorithm to predict acid taste attribute scores and (b) the gain of LVs.

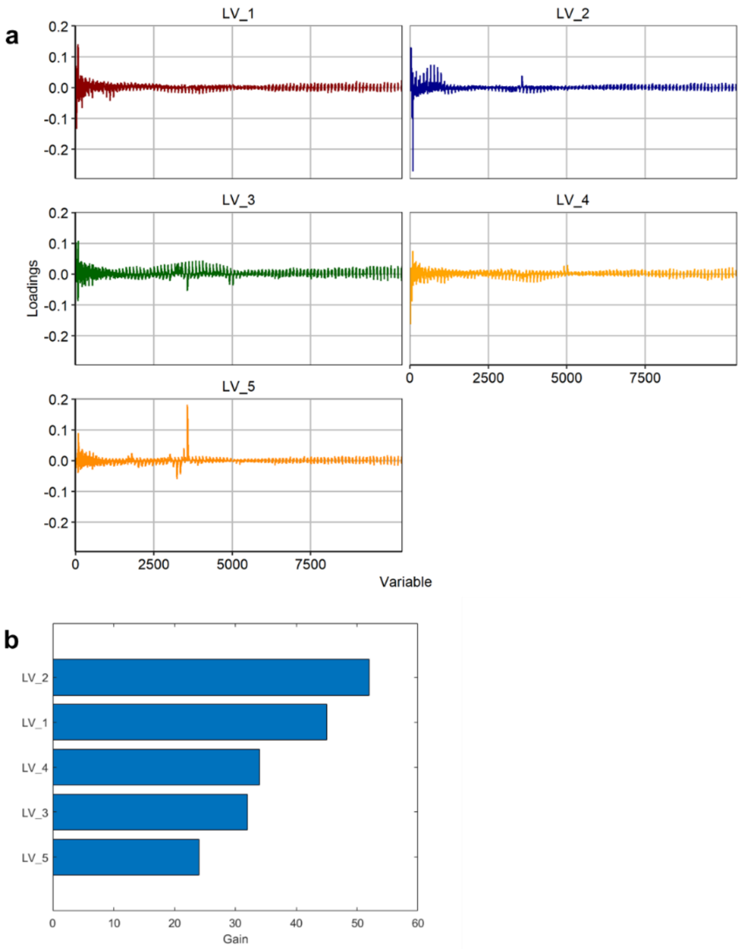

Figure S21. (a) A-TEEM variable loadings on PLS LVs 1-4 used in the XGB algorithm to predict bitter taste attribute scores and (b) the gain of LVs.

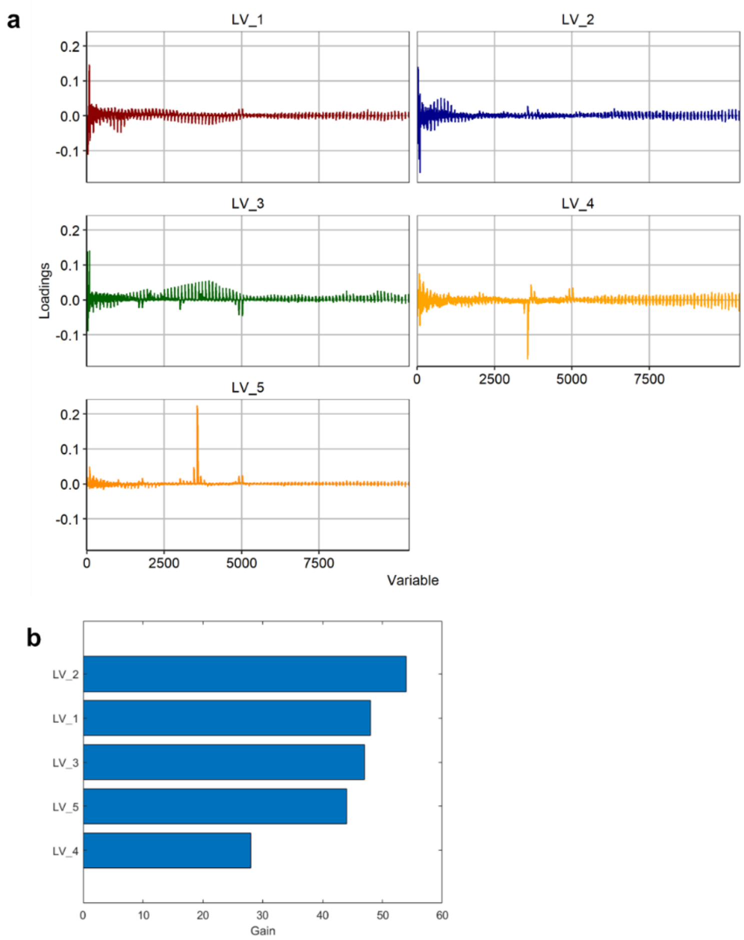

Figure S22. (a) A-TEEM variable loadings on partial least squares (PLS) regression latent variables (LVs) 1-4 used in the XGB algorithm to predict fruit sweetness taste attribute scores and (b) the gain of LVs.

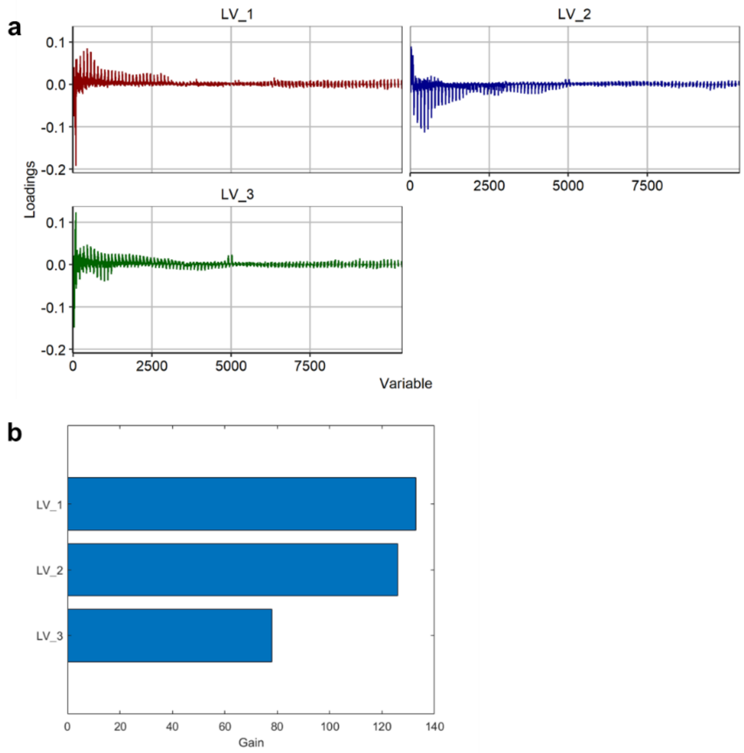

Figure S23. (a) A-TEEM variable loadings on PLS LVs 1-4 used in the XGB algorithm to predict alcohol intensity mouthfeel attribute scores and (b) the gain of LVs.

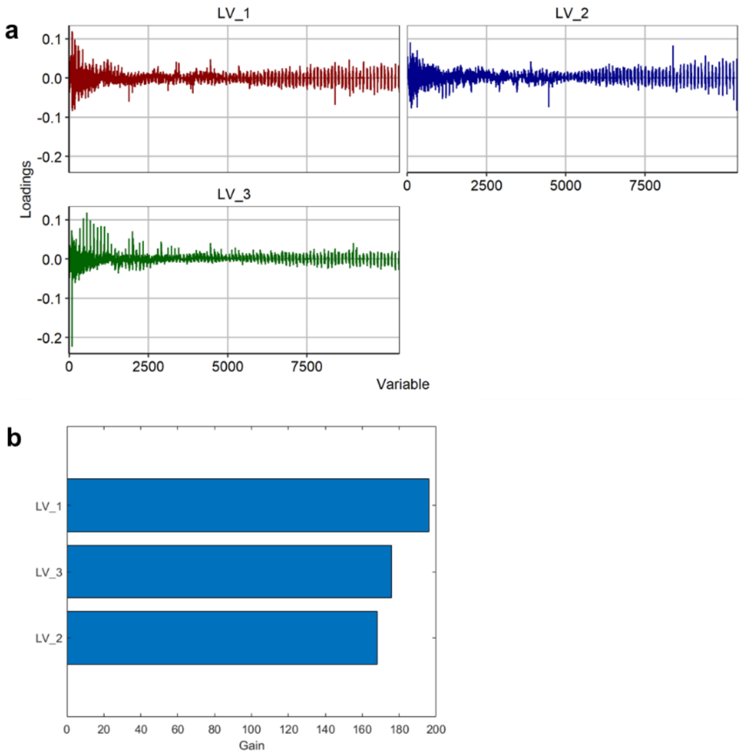

Figure S24. (a) A-TEEM variable loadings on partial least squares (PLS) regression latent variables (LVs) 1-4 used in the XGB algorithm to predict astringency mouthfeel attribute scores and (b) the gain of LVs.

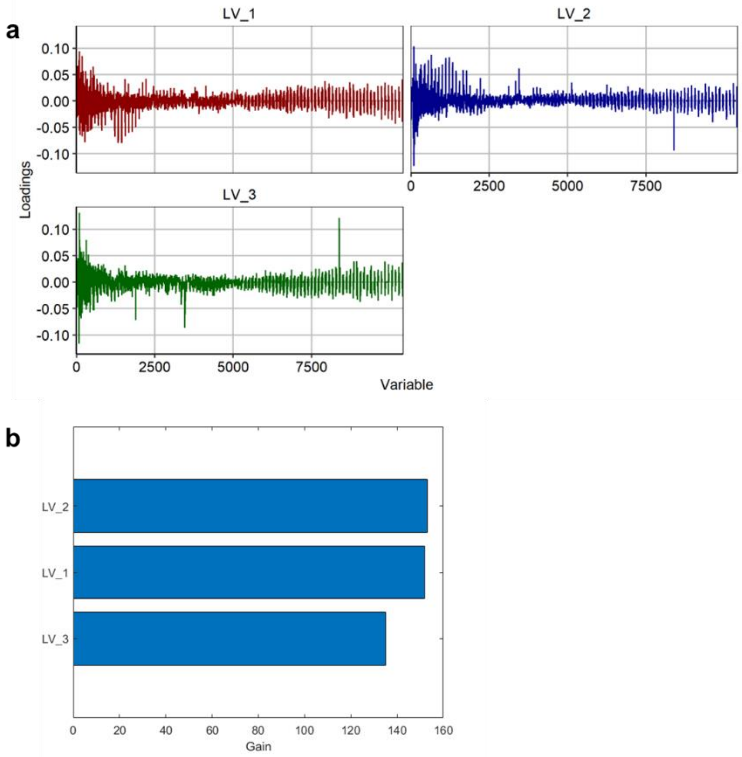

Figure S25. (a) A-TEEM variable loadings on PLS LVs 1-4 used in the XGB algorithm to predict body mouthfeel attribute scores and (b) the gain of LVs.

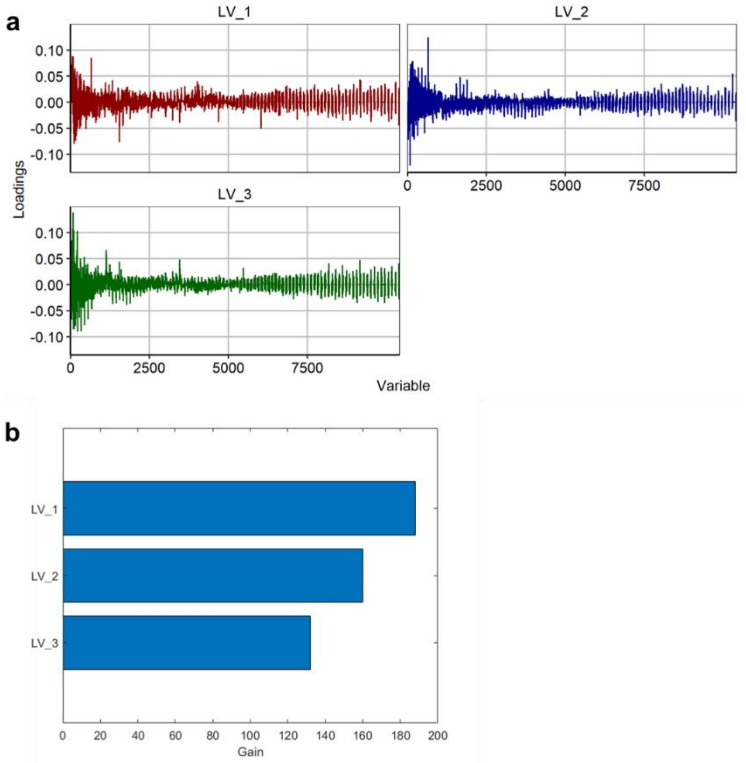

Figure S26. (a) A-TEEM variable loadings on partial least squares (PLS) regression latent variables (LVs) 1-4 used in the XGB algorithm to predict depth colour attribute scores and (b) the gain of LVs.

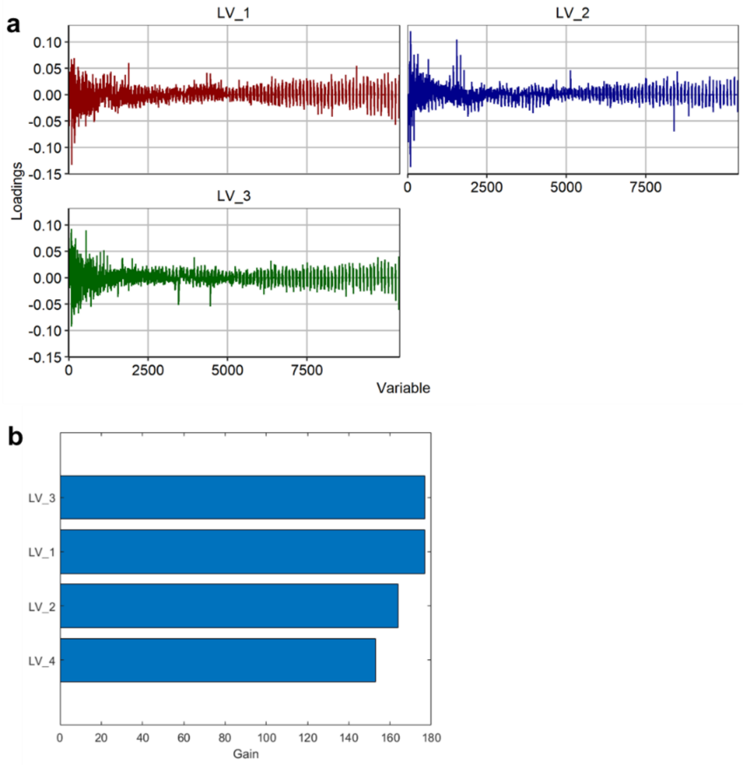

Figure S27. (a) A-TEEM variable loadings on PLS LVs 1-4 used in the XGB algorithm to predict hue colour attribute scores and (b) the gain of LVs.

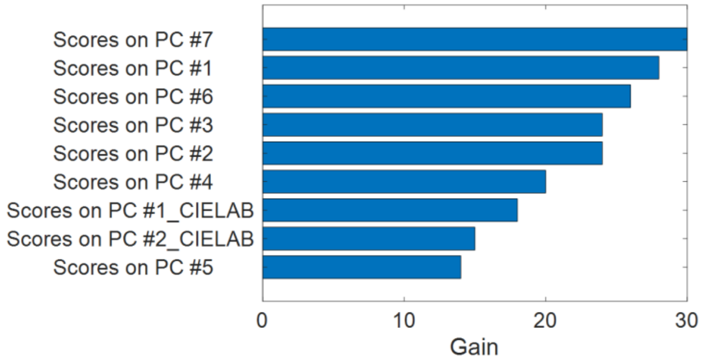

Figure S28. Gain of PC1-7 from principal component analysis (PCA) of A-TEEM data and PC1 and 2 from PCA of CIELAB data used to predict confectionery aroma.

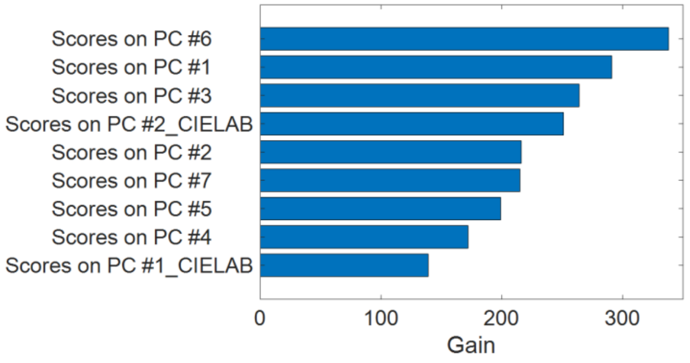

Figure S29. Gain of PC1-7 from PCA of A-TEEM data and PC1 and 2 from PCA of CIELAB data used to predict dark fruit aroma.

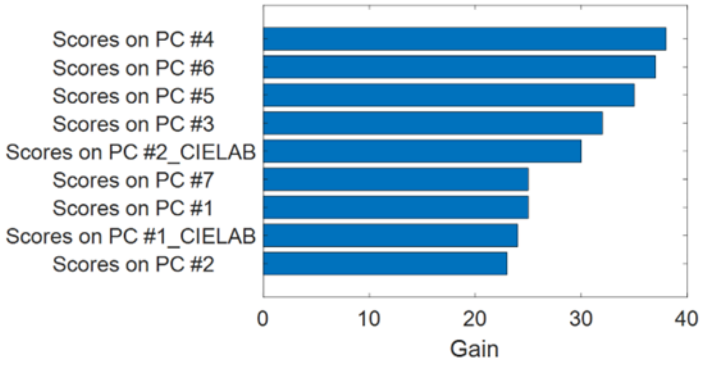

Figure S30. Gain of PC1-7 from PCA of A-TEEM data and PC1 and 2 from PCA of CIELAB data used to predict earthy fruit aroma.

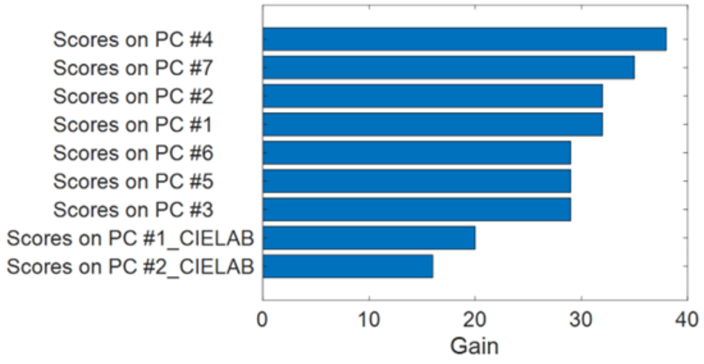

Figure S31. Gain of PC1-7 from PCA of A-TEEM data and PC1 and 2 from PCA of CIELAB data used to predict green fruit aroma.

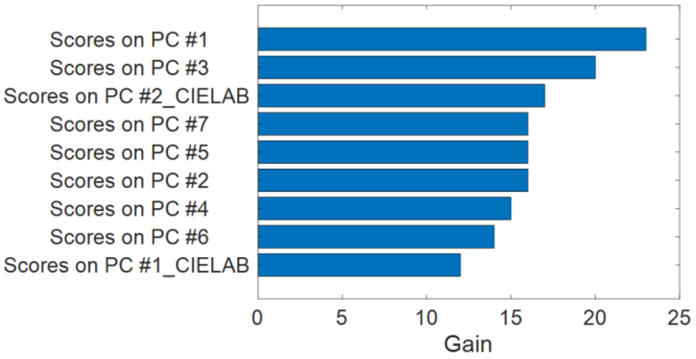

Figure S32. Gain of PC1-7 from principal component analysis (PCA) of A-TEEM data and PC1 and 2 from PCA of CIELAB data used to predict overall intensity aroma.

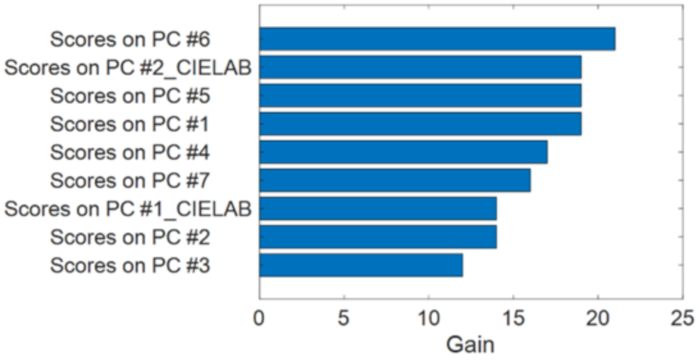

Figure S33. Gain of PC1-7 from PCA of A-TEEM data and PC1 and 2 from PCA of CIELAB data used to predict pepper aroma.

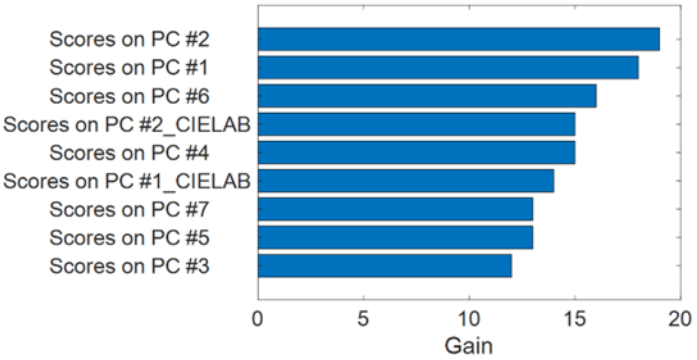

Figure S34. Gain of PC1-7 from PCA of A-TEEM data and PC1 and 2 from PCA of CIELAB data used to predict red fruit aroma.

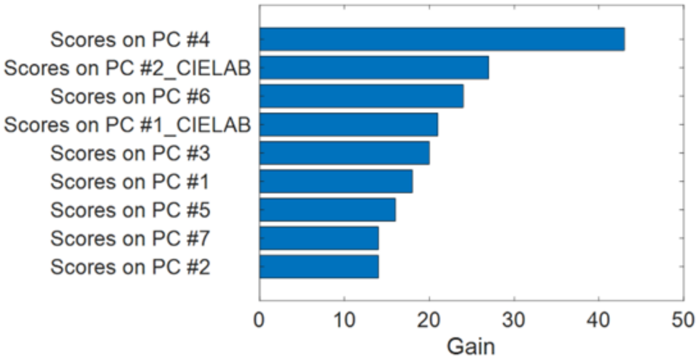

Figure S35. Gain of PC1-7 from PCA of A-TEEM data and PC1 and 2 from PCA of CIELAB data used to predict savoury aroma.

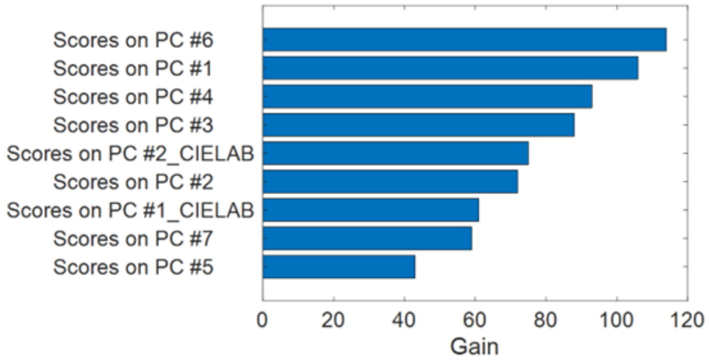

Figure S36. Gain of PC1-7 from principal component analysis (PCA) of A-TEEM data and PC1 and 2 from PCA of CIELAB data used to predict confectionery flavour.

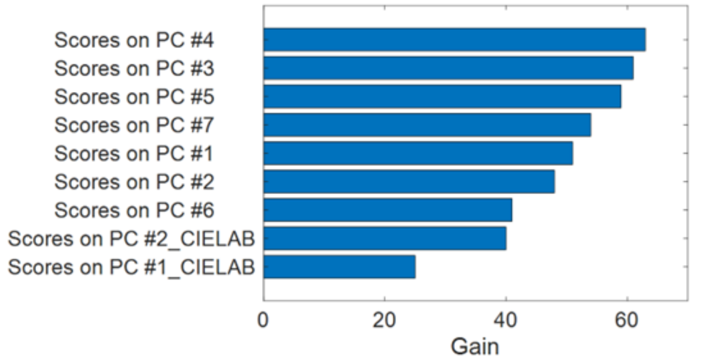

Figure S37. Gain of PC1-7 from PCA of A-TEEM data and PC1 and 2 from PCA of CIELAB data used to predict dark fruit flavour.

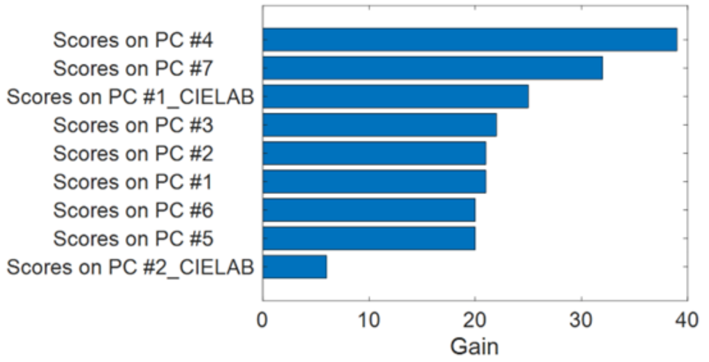

Figure S38. Gain of PC1-7 from PCA of A-TEEM data and PC1 and 2 from PCA of CIELAB data used to predict green flavour.

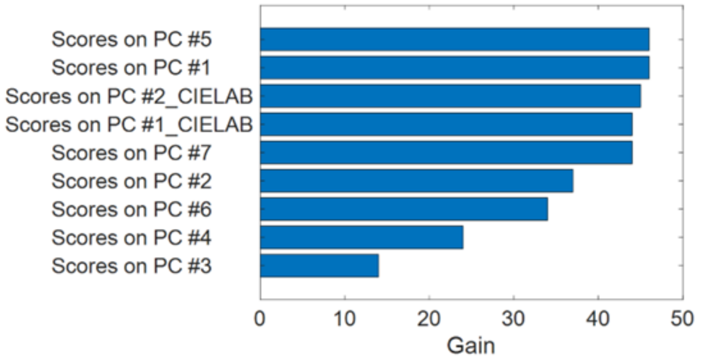

Figure S39. Gain of PC1-7 from PCA of A-TEEM data and PC1 and 2 from PCA of CIELAB data used to predict pepper flavour.

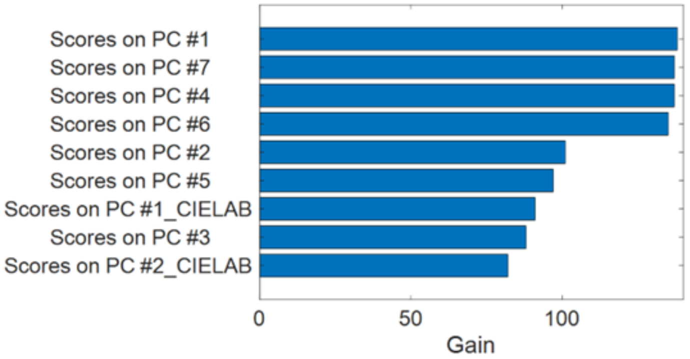

Figure S40. Gain of PC1-7 from principal component analysis (PCA) of A-TEEM data and PC1 and 2 from PCA of CIELAB data used to predict red fruit flavour.

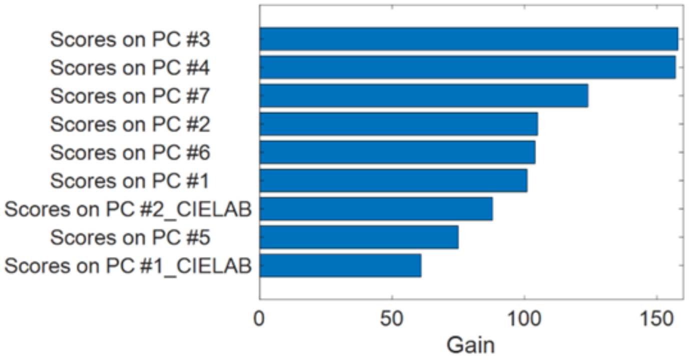

Figure S41. Gain of PC1-7 from PCA of A-TEEM data and PC1 and 2 from PCA of CIELAB data used to predict savoury flavour.

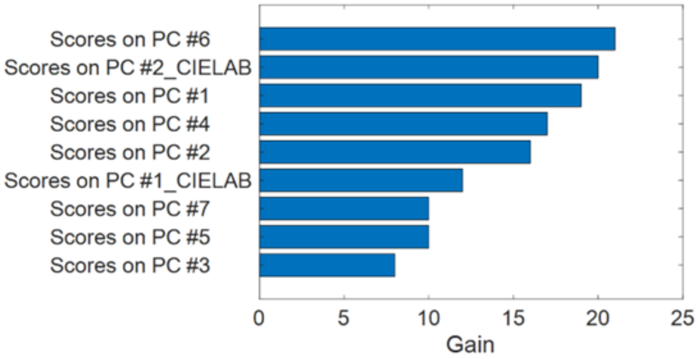

Figure S42. Gain of PC1-7 from PCA of A-TEEM data and PC1 and 2 from PCA of CIELAB data used to predict acid taste.

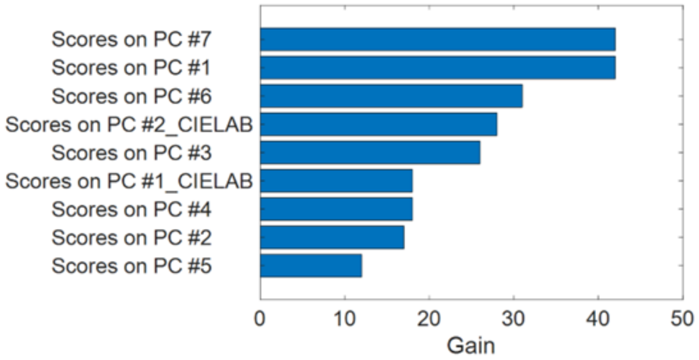

Figure S43. Gain of PC1-7 from PCA of A-TEEM data and PC1 and 2 from PCA of CIELAB data used to predict bitter taste.

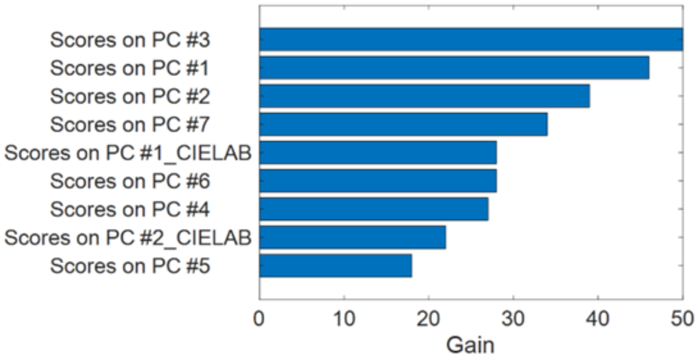

Figure S44. Gain of PC1-7 from principal component analysis (PCA) of A-TEEM data and PC1 and 2 from PCA of CIELAB data used to predict fruit sweetness taste.

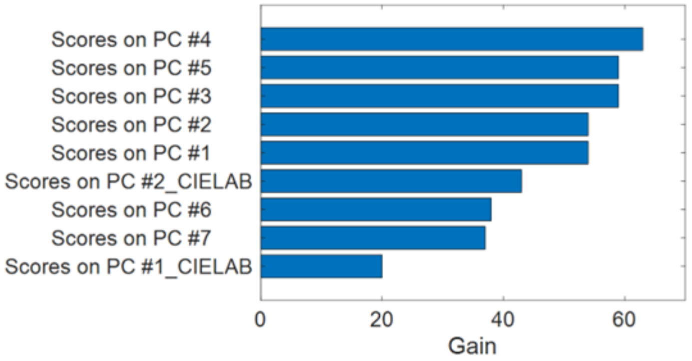

Figure S45. Gain of PC1-7 from PCA of A-TEEM data and PC1 and 2 from PCA of CIELAB data used to predict alcohol intensity mouthfeel.

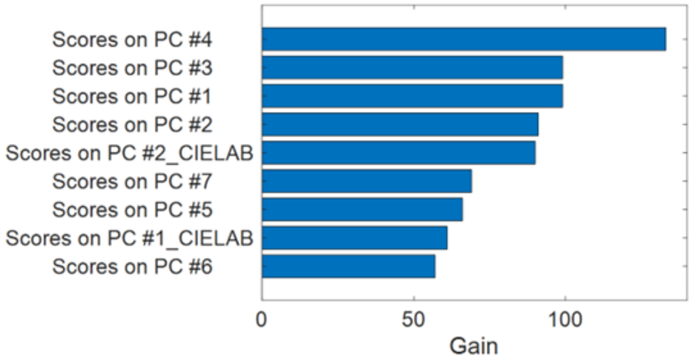

Figure S46. Gain of PC1-7 from PCA of A-TEEM data and PC1 and 2 from PCA of CIELAB data used to predict astringency mouthfeel.

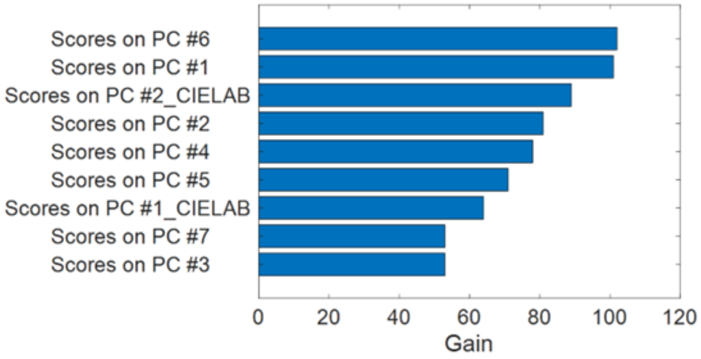

Figure S47. Gain of PC1-7 from PCA of A-TEEM data and PC1 and 2 from PCA of CIELAB data used to predict body mouthfeel.

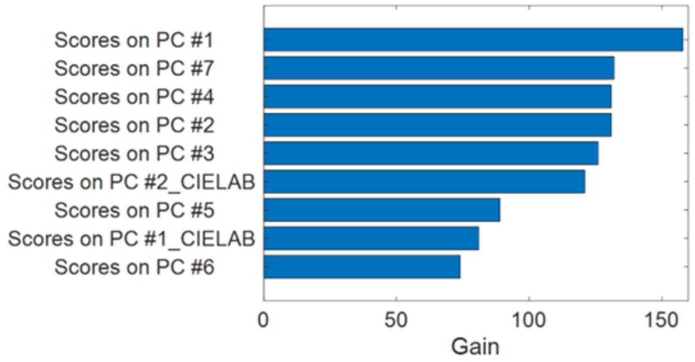

Figure S48. Gain of PC1-7 from principal component analysis (PCA) of A-TEEM data and PC1 and 2 from PCA of CIELAB data used to predict depth colour.

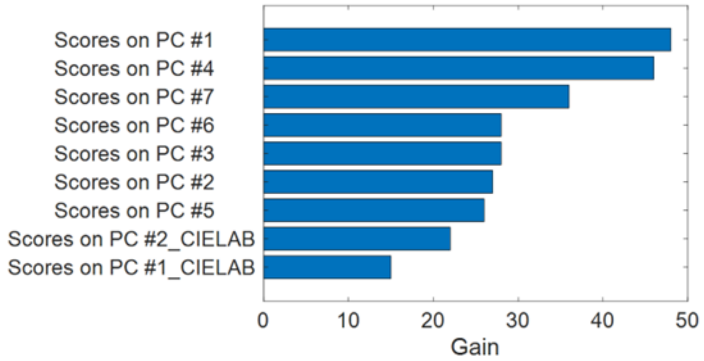

Figure S49. Gain of PC1-7 from PCA of A-TEEM data and PC1 and 2 from PCA of CIELAB data used to predict hue colour.
